# A novel role for GSK3β as a modulator of Drosha microprocessor activity and MicroRNA biogenesis

**DOI:** 10.1093/nar/gkw938

**Published:** 2016-10-23

**Authors:** Claire E. Fletcher, Jack D. Godfrey, Akifumi Shibakawa, Martin Bushell, Charlotte L. Bevan

**Affiliations:** 1 Imperial Centre for Translational and Experimental Medicine, Department of Surgery & Cancer, Imperial College London, Hammersmith Hospital, Du Cane Road, London, W12 0NN, UK; 2 Medical Research Council Toxicology Unit, Hodgkin Building, Lancaster Road, Leicester, LE1 9HN, UK

## Abstract

Regulation of microRNA (miR) biogenesis is complex and stringently controlled. Here, we identify the kinase GSK3β as an important modulator of miR biogenesis at Microprocessor level. Repression of GSK3β activity reduces Drosha activity toward pri-miRs, leading to accumulation of unprocessed pri-miRs and reduction of pre-miRs and mature miRs without altering levels or cellular localisation of miR biogenesis proteins. Conversely, GSK3β activation increases Drosha activity and mature miR accumulation. GSK3β achieves this through promoting Drosha:cofactor and Drosha:pri-miR interactions: it binds to DGCR8 and p72 in the Microprocessor, an effect dependent upon presence of RNA. Indeed, GSK3β itself can immunoprecipitate pri-miRs, suggesting possible RNA-binding capacity. Kinase assays identify the mechanism for GSK3β-enhanced Drosha activity, which requires GSK3β nuclear localisation, as phosphorylation of Drosha at S^300^ and/or S^302^; confirmed by enhanced Drosha activity and association with cofactors, and increased abundance of mature miRs in the presence of phospho-mimic Drosha. Functional implications of GSK3β-enhanced miR biogenesis are illustrated by increased levels of GSK3β-upregulated miR targets following GSK3β inhibition. These data, the first to link GSK3β with the miR cascade in humans, highlight a novel pro-biogenesis role for GSK3β in increasing miR biogenesis as a component of the Microprocessor complex with wide-ranging functional consequences.

## INTRODUCTION

MicroRNAs, first identified in 1993, are 18–22 nucleotide non-coding RNAs. The accepted dogma is that they negatively regulate gene expression through association with complementary sequences within target gene 3΄UTRs, leading to transcript degradation and/or translational inhibition (1,2). A single transcript can be targeted by hundreds of miRs, and individual miRs can target hundreds of genes, hence the regulatory activity of miRs is being increasingly accepted as a complex network of tissue-and disease-specific interactions (3,4). MiRs are transcribed by RNA polymerase II, generating a primary microRNA transcript (pri-miR), which is then 5΄ capped and adenylated (5). The majority of pri-miRs are polycistronic and generate several functional mature miRs. The pri-miR is cleaved into one or more ∼70 nt hairpin-structured precursor miRs (pre-miRs), by the Drosha-containing Microprocessor (MP) complex (6). Drosha, an RNase III enzyme, is stabilised by association with double-stranded RNA binding domain protein DiGeorge Critical Region 8 (DGCR8)/Partner of Drosha (Pasha) (7). Other cofactors such as p72, p68, FUS and hnRNPA1 modulate fidelity, efficiency and specificity of cleavage or act as scaffold proteins to aid complex formation (8). Some cofactors alter biogenesis of the entire miRNAome, others demonstrate activity against a defined miR subset. Thus, the MP is very large multi-protein complex (>650 kD in human cells (9)) containing at least 20 different polypeptides. Drosha cleavage generates a 2 nt 3΄ overhang, vital both for recognition by Exportin-5, which facilitates Ran-GTP-dependent export of the pre-miR to the cytoplasm, and for cleavage of the stem-loop by a second RNase III enzyme, Dicer (10,11). Optimal Dicer activity requires the accessory dsRBD protein TRBP/PACT, and yields a ∼22 nt miR duplex. The two strands separate and one strand associates with Argonaute-2 (AGO2), a protein component of the RNA-induced silencing complex (RISC). The mature miR guides RISC to complementary sequences within the 3΄UTR of target mRNAs, resulting in translational repression and/or transcript degradation.

MiR biogenesis is emerging as a stringently controlled and remarkably complex pathway, about which much remains to be learnt. Coordinated regulation, including feedback from miR targets, likely serves to prevent mis-expression of miRs both spatially and temporally, safeguarding sophisticated transcriptional processes. MiR processing is thought to be particularly important in development and tumourigenesis. For example, in early development many pri-miRs are expressed but not efficiently converted into their mature forms (12). Equally, reduced processing has been shown to contribute to widespread downregulation of many miRs in human cancers (11,13,14).

Glycogen synthase kinase 3β (GSK3β) is a serine/threonine protein kinase, initially identified as a regulator of glycogen metabolism, that has been shown to perform vital roles in a number of essential cellular signalling pathways, including Wnt/β-catenin, Hedgehog, Notch and Insulin signalling (15). It plays a key role in signal transduction during processes such as cell cycle progression, proliferation and inflammation. GSK3β phosphorylates diverse target proteins, and is itself regulated by phosphorylation. Its activity is decreased by Ser^9^ phosphorylation, mediated by the serine/threonine kinase Akt (a component of the PI3K/MAPK signalling pathway), MAPK-activated protein kinase-1 or p70 ribosomal S6 kinase-1. In contrast, phosphorylation at Tyr^216^ results in activation of GSK3β and is believed to be vital for signal transduction in resting cells (16). It has been established that more than 40 proteins are substrates for GSK3β (17), including cyclin D1 (18) and the transcription factors AP1, NFκB, c-Jun, GR and Notch (19–21), permitting highly sensitive regulation of cell cycle progression in response to extracellular stimuli.

GSK3β initially gained prominence as a drug target in treatment of diabetes mellitus and obesity (22,23). It also plays important roles in signal transduction in several key neurotransmitter pathways so is linked to mood disorders, bipolar disorder, depression and schizophrenia, and the GSK3β inhibitors lithium and valproate are currently used to treat such conditions (24,25). In cancer, GSK3β has been implicated in development and progression of breast (26), brain (27), pancreatic (28), colon (29) and prostate tumours (30), although it displays disparate activity in differing tissues and tumour types. In breast cancer cells, activation of GSK3β by rapamycin induces downregulation of cyclin D1, cell cycle arrest and inhibition of anchorage-dependent growth (26). GSK3β may also play a role in preventing epithelial to mesenchymal transition (EMT) in tumourigenesis, as its inhibition promotes EMT in cultured epithelial cells (31). In contrast, GSK3β overexpression has been observed in ovarian, colon and pancreatic tumours (21) resulting in enhanced proliferation and survival of ovarian cancer cells *in vivo* and *in vitro* (32). Additionally, GSK3β inhibition suppressed ovarian cancer cell proliferation *in vitro* (32) and decreased growth and survival of colon cancer cells *in vivo* (29).

It was recently shown that inhibition of GSK3β using small molecule inhibitors decreases levels of the majority of mature miRs in mouse embryonic stem cells, the data suggesting a reduction in nuclear Drosha levels in such cells may be responsible (33). It has also been reported that GSK3β phosphorylates Drosha at residues S^300^ and S^302^ (34). Impacts of such modifications, and of GSK3β inhibition, on Drosha's essential ribonuclease activity and miR biogenesis were, until now, unknown. The data presented here are the first to describe a mechanism for GSK3β regulation of miR biogenesis as a regulatory component of the MP. We demonstrate that GSK3β enhances Drosha association with its cofactors, DGCR8 and p72 and increases Drosha:pri-miR binding to enhance pri-miR cleavage. This is achieved through direct binding of GSK3β to DGCR8 and p72 within the Microprocessor in an RNA-dependent manner. In addition, we have shown that GSK3β-mediated phosphorylation of Drosha at S^300^ and S^302^ increases miR biogenesis not through altered Drosha localisation, but by enhancing Drosha:DGCR8 interaction. We hypothesise that GSK3β constitutes a ‘missing link’ between essential mitogenic signalling pathways and miR biogenesis.

## MATERIALS AND METHODS

### Mammalian cell culture

Cells were maintained at 37°C in 5% CO_2_. HeLa, HEK293T and COS-1 cells were maintained in Dulbecco's Modified Eagle's Medium (Sigma), LNCaP and PC3 cells were maintained and passaged in RPMI-1640 (Sigma). All media supplemented with 10% fetal bovine serum, 100 U/ml penicillin, 100 μg/ml streptomycin and 2 mM L-glutamine (Sigma).

### Cell lysis, Western blotting and antibodies

Cells were lysed and protein extracted as described (35). Proteins were resolved by 8–12% SDS-polyacrylamide gel electrophoresis and electroblotted to nitrocellulose membrane (Bio-Rad). After blocking (5% non-fat dried milk powder in 0.05% Tween-20 in 1xPBS, or 5% BSA in TBST for phospho-proteins) for 40 min, membranes were incubated with rabbit anti-Drosha pAb (Sigma, SAB4200151), mouse anti-GSK3β mAb (Abcam, ab93926), rat anti-AGO2 Ab (gift from Geok Tan, Imperial College London), mouse anti-FUS mAb (SantaCruz, sc-47711), mouse anti-Flag epitope tag M2 mAb (Sigma, F1804), rabbit anti-DGCR8 pAb (Sigma, SAB4200089), rabbit anti-DGCR8 pAb (Abcam ab36865), mouse anti-HA mAb (Covance, 16B12), mouse anti-phospho-serine (Sigma P5747), rabbit anti-PTEN pAb (R+D systems, AF847), rabbit anti-FOXO1 mAb (Abcam, ab52857), rabbit anti-ZEB1 mAb (Cell Signalling, 3396), mouse anti-β-tubulin mAb (Sigma, T4126) or mouse anti-β-actin mAb (Abcam, ab6276) for 1 h and visualised using goat anti-mouse, goat anti-rabbit or goat anti-rat IgG-HRP as appropriate. Detection was by Luminata Forte HRP substrate (Millipore).

### Plasmid stocks

pCK-Flag-Drosha construct was a kind gift from Prof. V. Narry Kim, Seoul University. pCK-Flag-Drosha-S^300^A,S^302^A and pCK-Flag-Drosha-S^300^E,S^302^D plasmids were generated by site-directed mutagenesis of the pCK-Flag-Drosha vector using the QuikChange Lightning Site-Directed Mutagenesis Kit (Stratagene). pMT23-c-Myc-GSK3β-S^9^A and pMT23-HA-GSK3β-K^85^R plasmids were generously gifted by Dr Robert Kypta, Imperial College London. pMT23-HA-GSK3β (wild-type) and pMT23-HA-GSK3β-S^9^A plasmids were generated by site-directed mutagenesis of the pMT23-HA-GSK3β-K^85^R vector as above. CMV-pri-miR-23a27a24-2-GL4.18 Drosha activity reporter vector was constructed as previously described (36). pGEMT Easy-pri-miR-23a27a24-2 was generated by insertion of the genomic miR-23a27a24-2 sequence into the multiple cloning site. pMiRTarget-ACLY 3΄UTR was a kind gift of Dr Hector Keun (Imperial College London), generated by insertion of the full-length ACLY 3΄UTR 3΄ of the luciferase gene in the pMiRTarget vector. pMiRTarget-ACLY 3΄UTR miR-27a binding site (BS) mutant was generated by G→U point mutation of the pMiRTarget-ACLY 3΄UTR vector at nucleotide 700 of the ACLY 3΄UTR. This nucleotide is located in the centre of the miR-27a seed region binding site (Supplementary Figure S9).

### Drosha cleavage activity reporter luciferase assays

The CMV-pri-miR-23a27a24-2-GL4.18 Drosha activity reporter vector was cotransfected into Cos-1 or HEK293T cells alongside pMT23-HA-GSK3β-S^9^A or pMT23-HA-GSK3β-K^85^R mutant GSK3β expression vectors, or pCK-Flag-Drosha or pCK-Flag-Drosha-S^300^E,S^302^D Drosha expression vectors as appropriate using the calcium phosphate method (37). Twenty-four hours post-transfection, cells were treated with the GSK3β inhibitor, 99021, if required and harvested after a further 24 h. Luciferase assays were performed using the Luclite assay (Packard, USA) and activity normalised for transfection efficiency using the Galacton kit (Tropix) as previously described (38).

### MiR, pre-miR, pri-miR and mRNA quantitative real-time PCR

Mature miR expression was quantified by quantitative real-time RT-PCR using TaqMan microRNA assays and TaqMan Universal PCR Master Mix (Applied Biosystems) according to the manufacturer's protocol. For detection of pri-miRs, Drosha coding region and ACLY, ZEB1, PTEN or FOXO1 3΄UTRs, cDNA was prepared from 500 ng total RNA using Precision qScript Reverse Transcription kit (PrimerDesign) and oligo d(T) primers. cDNAs were amplified using either (i) 2x Fast SYBR Green Master Mix (Applied Biosystems) and 250 nM forward and reverse primers (see Supplementary Table S1), or (ii) TaqMan pri-miR assays (Applied Biosystems) for pri-miR-23a27a24-2, pri-miR-141/200c and pri-miR-182 and 2x Taqman Fast Universal PCR Master Mix No AmpErase UNG as per manufacturer's instructions. For detection of pre-miRs, small RNAs (<200 nt) were isolated using the miRVana MiRNA Isolation Kit (Ambion). A total of 500 ng RNA was reverse transcribed using Precision qScript Reverse Transcription kit (PrimerDesign) and random nonamers. cDNAs were amplified using 2x Fast SYBR Green Master Mix (Applied Biosystems) and 250 nM forward and reverse primers to pre-miR-27a (see Supplementary Table S1). All data were analysed using the ΔΔC_t_ method, with U18 and L19 as endogenous references for miR and mRNA/pre-miR/pri-miR levels respectively, using DMSO/ethanol-treated samples as calibrators where appropriate.

### RNA-immunoprecipitation

Full protocol is described in Supplementary Methods. Briefly, lysates were generated from HEK293T cells expressing exogenous GSK3β mutants and Flag-Drosha and incubated with anti-Flag affinity gel (Sigma) overnight at 4°C with rotation. Beads were washed, reconstituted with DNase solution (Qiagen) and treated with proteinase K. RNA was then extracted from beads using Trizol LS (Life Technologies) according to manufacturer's instructions and qRT-PCR performed for pri-miRs.

### Immunofluorescent cell staining

Cos-1, PC3 or LNCaP cells on coverslips were fixed with 1% formaldehyde in PBS at room temperature for 10 min, washed in PBS and blocked with 10% goat serum in PBS for 1 h. Primary antibodies (Rb anti-Drosha, Rb anti-DGCR8, Ms anti-HA and Ms anti-GSK3β) were diluted 1/100-1/200 in 10% goat serum and added to cells for 1 h at room temperature, as appropriate. Following washing with PBS, secondary antibodies (Alexa Fluor 488 Goat anti-Mouse and 594 Goat anti-Rabbit SFX kits, Invitrogen) were diluted 1/200 in 10% goat serum and added to cells for 1 h at room temperature in the dark. Cells were washed briefly in PBS and coverslips were mounted onto glass slides using DAPI-containing Vectashield mounting solution. Staining was visualised using a Zeiss LSM510 confocal microscope.

### Flag-Drosha subcellular fractionation

Subcellular fractionation was performed as described (39) to isolate cytoplasmic, soluble nuclear and chromatin-bound protein fractions. See Supplementary Methods.

### Immunoprecipitation

Immunoprecipitation was performed as described in the Supplementary Methods. Briefly, lysates were generated from HEK293T cells expressing exogenous pMT23-GSK3β-WT/ S^9^A/ K^85^R/ K^85^A,K^86^A and/or Flag-Drosha-WT/S^300^A,S^302^A/S^300^E,S^302^D as appropriate, pre-cleared and incubated with anti-Flag M2 or EZView anti-HA agarose beads (Sigma) as appropriate at 4°C overnight with rotation. Beads were washed in TBS, followed by RNase A treatment (200 μg/ml) for 15 min at 4°C, as appropriate. Beads were boiled in IP sample loading buffer, pelleted and supernatant subjected to Western blotting. For immunoprecipitation of endogenous proteins, lysates were generated from 99021-treated HEK293T cells and incubated with rabbit anti-Drosha (Cell Signalling, 3364) or mouse anti-GSK3β (Abcam, ab93926) antibody-bound Protein G Dynabeads (ThermoFisher) or anti-phosphoserine–agarose (Sigma-Aldrich, A8076) as appropriate at 4°C overnight with rotation. Beads were washed x3 with Wash Buffer (Dynabeads® Protein G Immunoprecipitation Kit, ThermoFisher), eluted as above and subjected to Western blotting.

### *In vitro* pri-miR processing assay

*In vitro* processing assays were performed as described (40) with modifications. Briefly, pGemT Easy vector containing 652 bp pri-miR-23a27a24-2 sequence was linearised at a *SalI* restriction site ∼30 bp 3΄ of the end of the pri-miR sequence. The pri-miR was *in vitro* transcribed from the T7 promoter using MEGAscript T7 *in vitro* transcription kit (Ambion) and 0.75 μl of α-32P-UTP (40 μCi/μl, 800 mCi/mmole, Perkin Elmer NEG007C001MC), followed by phenol/chloroform extraction. Precipitated RNA was resolved on a 6% acrylamide:urea gel, which was exposed to film. α-32P-UTP-labelled pri-miR-23a27a24-2 was cut from the gel and eluted in 0.3 M sodium acetate (pH 5.5), 2% SDS. *In vitro* processing was performed by incubation (37°C, 90 min) of α-32P-UTP-labelled pri-miR-23a27a24-2 with Flag-Drosha immunoprecipitated from HEK293T cells transfected with pCK-Flag-Drosha ± pMT23-HA-GSK3β-WT/S9A/K85R for 48 h. RNA was phenol/chloroform-extracted and separated on 6% and 12.5% acrylamide:urea gels alongside Decades RNA Markers (ThermoFisher), which were prepared according to the manufacturer's protocol. The gel was exposed to film overnight at −80°C in a cassette with intensifying screen.

### *In vitro* kinase assay

Twenty five amino acid peptides were synthesised corresponding to WT Drosha 289–313 (RERHRHRDNRR**S**P**S**LERSYKKEYKR) or S^300^A,S^302^A Drosha 289–313 (RERHRHRDNRR**A**P**A**LERSYKKEYKR), containing predicted GSK3β phosphorylation sites. Twenty five micrograms of the above Drosha peptides were incubated with 150 ng GSK3β, 10 μM cold ATP and 5 μl (γ-32P)-ATP (10 Ci/mmol, 2 mCi/ml, Perkin Elmer) in 50 μl kinase assay buffer (60 mM HEPES-NaOH pH7.5, 3 mM MgCl_2_, 3 mM MnCl_2_, 3 μM sodium orthovanadate, 1.2 mM DTT, 0.05 μg/μl PEG20.000) for 1 h at 30°C. Reactions were terminated by incubation at 65°C for 20 min. A total of 10 μl of each reaction was dotted onto nitrocellulose membrane, left to dry for 2–3 min, washed x3 with TBST and exposed to X-ray film.

### ACLY 3΄UTR luciferase reporter assay

pMiRTarget-ACLY 3΄UTR WT or pMiRTarget-ACLY 3΄UTR miR-27a binding site (BS) mutant were transfected into HEK293T cells alongside the pdmLacZ β-galactosidase reporter plasmid using the calcium phosphate method (37). Twenty-fours hours post-transfection, cells were treated with 99021 (2 μM) and harvested after a further 24 h. Luciferase assays were performed using the Luclite assay (Packard, USA) and activity normalised for transfection efficiency using the Galacton kit (Tropix) as previously described (38).

### Statistical analysis

Normally distributed continuous variables were assessed by Student‘s *t*-test. Strength of correlation between two normally distributed continuous variables was assessed by Pearson's correlation coefficient (r). *P* ≤ 0.05 was interpreted to denote statistical significance.

## RESULTS

### Inhibition of GSK3β reduces MiR biogenesis through inhibition of Drosha activity and repression of pri-MiR processing

**Figure 1.**
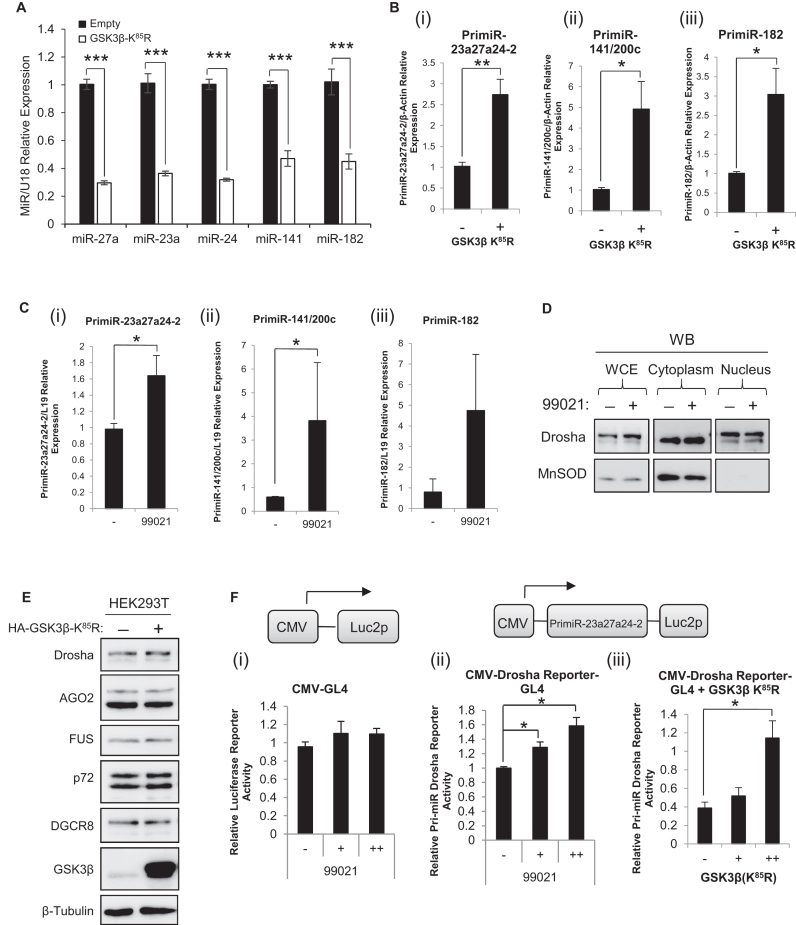
Inhibition of GSK3β reduces MiR biogenesis through repression of pri-MiR processing. (**A**) qRT-PCR analysis of miR-27a, miR-23a, miR-24, miR-141 and miR-182 levels in HEK293T cells transfected with pMT23-HA-GSK3β(K^85^R) for 48 h. U18 was used as a normalisation gene. (**B** and **C**) qRT-PCR analysis of pri-miR-23a27a24-2 (i), pri-miR-141/200c (ii) and pri-miR-182 (iii) expression from HEK293T cells either (**B**) transfected with pMT23-HA-GSK3β(K^85^R), or (**C**) treated with 99021 (2 μM) for 48 h. β-Actin was used as normalisation gene. (**A, B** and **C**) Columns: mean ± SEM for three independent experiments performed in triplicate. (**D**) Western blot analysis of Drosha and MnSOD protein levels in cytoplasmic and nuclear fractions of LNCaP cells treated with 99021 for 48 h. MnSOD was used as a cytoplasmic control. A representative blot of three independent experiments is shown. (**E**) Western blot analysis of Drosha, AGO2, FUS, p72 and DGCR8 protein levels in HEK293T cells transfected with pMT23-HA-GSK3β(K^85^R) for 48 h. β-Tubulin was used as a loading control and for normalisation. A representative blot of three independent experiments is shown. (**F**) Luciferase activity in extracts of COS-1 cells transfected with 250 ng (i) CMV-GL4 or (ii,iii) CMV-Pri-miR-23a27a24-2-GL4, and treated with (i,ii) 99021 (0,1,2 μM), or co-transfected with 0, 100 or 400 ng (iii) pMT23-HA-GSK3β(K^85^R) for 48 h. Luciferase was normalised for transfection efficiency (β-galactosidase activity) and mean ± SEM of three independent experiments performed in duplicate is shown. *P ≤ 0.05, **P ≤ 0.005, ***P ≤ 0.0001. See also Supplementary Figure S1.

As GSK3β interacts with and modulates the localisation of Drosha (34,41), we hypothesised that GSK3β expression and/or activity may alter pri-miR processing, and ultimately mature miR levels. To address this, a number of cell lines were treated with the highly potent and specific GSK3β inhibitor, 6-(2-(4-(2,4-Dichloro-phenyl)-5-(4-methyl-1H-imidazol-2-yl)-pyrimidin-2-ylamino)-ethylamino)-nicotinonitrile (known as CT 99021 - CHIR 99021 and referred to as 99021 throughout) (22), or transfected with a dominant-negative form of GSK3β, GSK3β-K^85^R (42,43) to control for possible off-target effects of 99021. GSK3β-K^85^R transfection reduced levels of mature miR-27a, −23a, −141 and −182 by up to 70% in HEK293T cells (Figure 1A). The same effect was observed in LNCaP prostate cancer cells following GSK3β-K^85^R transfection (Supplementary Figure S1A), and was corroborated by a 50% reduction in levels of the same mature miRs following 99021 treatment of LNCaP cells (Supplementary Figure S1B). Further, levels of corresponding pri-miRs were increased by up to 4-fold following GSK3β-K^85^R transfection or 99021 treatment of HEK293T cells (Figure 1B and C); significantly increased pri-miR levels were also observed following GSK3β-K^85^R transfection of HeLa cells (Supplementary Figure S1C).

As it has been previously demonstrated that GSK3β can phosphorylate Drosha and alter its subcellular localisation (34,41), it was possible that the effects of GSK3β inhibition on miR maturation may simply be attributable to altered Drosha localisation. However, 99021 treatment of LNCaP cells followed by cell fractionation showed no alteration in nuclear Drosha protein levels (Figure 1D), refuting this hypothesis. Additionally, decreased miR levels are not attributable to altered protein levels of MP or RISC components, since transfection of GSK3β-K^85^R did not alter protein levels of Drosha, DGCR8, FUS, p72 or of the RISC component, AGO2 in HEK293T (Figure 1E) or HeLa cells (Supplementary Figure S1D). In addition, 99021 treatment of LNCaP prostate cancer cells did not alter Drosha protein levels (Supplementary Figure S1E). These data indicate that GSK3β can modulate miR biogenesis without altering abundance or localisation of key miR biogenesis pathway proteins.

Since GSK3β inhibition appears to inhibit pri-miR to mature miR processing, it was hypothesised that 99021-treatment may alter Drosha activity. To investigate this further, a reporter vector was generated in which the genomic miR-23a27a24-2 sequence is located 5΄ of the *luc2p* gene, under the control of a CMV promoter (Figure 1F). Transcription yields the pri-miR-23a27a24-2 linked to the luc2p transcript and Drosha-mediated cleavage of the pri-miR disrupts the transcript, resulting in loss of luciferase activity, so Drosha activity is inversely correlated with luciferase activity. This approach has been shown to be a sensitive assay for Drosha-mediated cleavage of a specific pri-miR species in previous studies (44). Cos-1 cells were transfected with the Drosha reporter, followed by treatment with 99021. A statistically significant 60% increase in luciferase activity was observed following 99021 treatment, indicating a 60% loss of Drosha activity upon GSK3β inhibition (Figure 1Fii). No alteration to luciferase activity was observed upon transfection of a Drosha reporter lacking a pri-miR sequence (Figure 1Fi). In addition, a significant dose-dependent increase in luciferase activity (reduction of Drosha activity) was observed following co-transfection of the Drosha reporter plasmid with increasing amounts of GSK3β-K^85^R (Figure 1Fiii). These data suggest that GSK3β regulates miR levels by modulating Drosha activity towards pri-miR substrates.

Taken together, these data represent the first evidence of a role for GSK3β in miR maturation in human cells. Our findings indicate that GSK3β inhibition reduces miR biogenesis by repressing Drosha activity towards pri-miRs, leading to accumulation of miR precursors. Thus, GSK3β may be a miR maturation-enhancing factor. This is in agreement with the demonstration by Wu *et al.* that 90.4% of differentially-regulated miRs were downregulated following 99021 treatment of mouse embryonic stem cells (33).

### Constitutively active GSK3β S^9^A mutant increases Drosha cleavage activity and enhances MiR biogenesis

**Figure 2.**
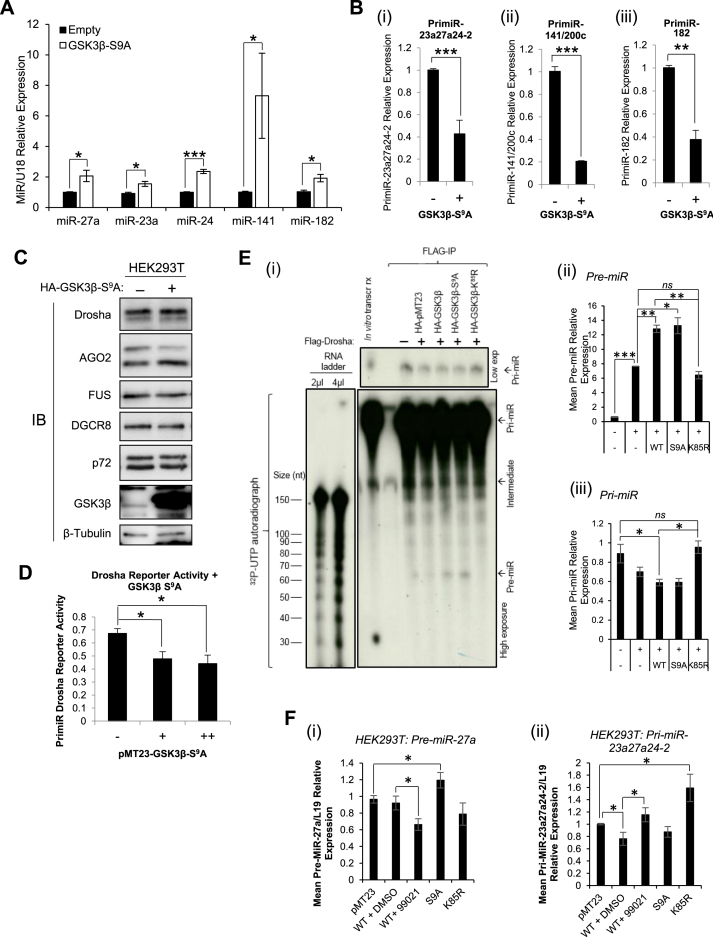
GSK3β activation enhances MiR biogenesis and GSK3β modulation alters pre-miR synthesis (**A**) qRT-PCR analysis of miR-27a, miR-23a, miR-24, miR-141 and miR-182 levels in HEK293T cells transfected with pMT23-HA-GSK3β(S^9^A) for 48 h. U18 was used as a normalisation gene. (**B**) qRT-PCR analysis of (i) pri-miR-23a27a24-2, (ii) pri-miR-141/200c and (iii) pri-miR-182 expression from HEK293T cells transfected with pMT23-HA-GSK3β(S^9^A) for 48 h. β-Actin was used as normalisation gene. (**A** and **B**) *Columns:* mean ± SEM for three independent experiments performed in triplicate. (**C**) Western blot analysis of Drosha, AGO2, FUS, DGCR8 and p72 protein levels in HEK293T cells transfected with pMT23-HA-GSK3β(S^9^A) for 48 h. β-Tubulin was used as a loading control. (**D**) Luciferase activity in extracts of COS-1 cells transfected with 250 ng CMV-Pri-miR-23a27a24-2-GL4 and 0, 100 or 400 ng pMT23-HA-GSK3β(S^9^A) for 48 h. Luciferase was normalised for transfection efficiency (β-galactosidase activity) and mean ± SEM of three independent experiments performed in duplicate is shown. (**E**) *In vitro* Drosha-mediated pri-miR processing assay analysis of pre-miR levels following incubation of *in vitro* transcribed radio-labelled pri-miR-23a27a24-2 with Microprocessor complex immunoprecipitated from HEK293T cells transfected with Flag-Drosha ± GSK3β-WT, -S^9^A or -K^85^R for 48 h. Products of *in vitro* processing reactions were resolved on a 6% acrylamide:urea gel and exposed to film. A representative image of (i) three independent experiments is shown. Densitometry was performed using Image J software (ii and iii)*. Columns:* mean ± SEM for three independent experiments. Images of biological replicate experiments and complete gel images can be found in Supplementary Figure S2B and S3. (**F**) qRT-PCR analysis of (i) pre-miR-27a and (ii) pri-miR-23a27a24-2 levels in HEK293T cells treated with either 2 μM 99021, an equal volume of DMSO or transfected with pMT23-HA-GSK3β(WT) pMT23-HA-GSK3β(S^9^A) or pMT23-HA-GSK3β(K^85^R) for 48 h. L19 was used as a normalisation gene. *Columns:* mean ± SEM for three independent experiments performed in duplicate. *P ≤ 0.05, **P ≤ 0.005, ***P ≤ 0.0001. See also Supplementary Figures S2 and S3.

To provide further evidence for the importance of GSK3β activity for miR maturation, converse experiments were performed using a vector expressing constitutively active GSK3β: GSK3β-S^9^A. Transfection of GSK3-S^9^A into HEK293T significantly increased levels of mature miR-27a, miR-23a, miR-24, miR-141 and miR-182 by up to 7.5-fold (Figure 2A), with similar effects also observed in HeLa cells (Supplementary Figure S2A). In contrast, levels of corresponding pri-miR precursors were significantly reduced by up to 80% in HEK293T cells following GSK3-S^9^A transfection (Figure 2B). As anticipated, GSK3β-S^9^A did not alter protein levels of MP and RISC components, including Drosha (Figure 2C), and showed comparable localisation to both WT and dominant-negative GSK3β upon transfection into Cos-1 and LNCaP cells (Supplementary Figure S4). However, expression of constitutively activate GSK3β in Cos-1 cells significantly reduced luciferase activity of the Drosha reporter vector by up to almost 40%, indicating enhanced Drosha activity in the presence of GSK3β-S^9^A (Figure 2D). These data support the hypothesis that active GSK3β promotes miR biogenesis by increasing Drosha-mediated cleavage of pri-miRs. If this is the case, GSK3β activity should increase, and its inhibition decrease, pre-miR levels. Thus, *in vitro* pri-miR processing assays were performed, in which *in vitro* transcribed and radio-labelled pri-miR-23a27a24-2 was incubated with Flag-Drosha MP complex immunoprecipitated from HEK293T cells post-transfection with WT, constitutively-active or dominant-negative GSK3β. As anticipated, no pre-miR was produced in the absence of Flag-Drosha (Figure 2E). Further, WT GSK3β enhanced production of pre-miRs in the presence of Drosha compared to Drosha alone (Figure 2E). S^9^A-GSK3β did not appear to increase pre-miR levels versus WT under these experimental conditions, however, addition of dominant-negative GSK3β-K^85^R significantly reduced pre-miR levels (Figure 2E). Pre-miR bands were observed in alignment with the 60 nt RNA marker (Figure 2E and Supplementary Figure S3), which is consistent with expected pre-miR-23a, −27a and −24 sizes of 57 nt, 62 nt and 59 nt, respectively. Additional experimental replicates are shown (Supplementary Figure S2B) and Western blotting was performed on input cell lysates to demonstrate equal expression of the GSK3β and Flag-Drosha constructs (Supplementary Figure S2C). Uncropped images of different exposure lengths from one biological replicate are shown (Supplementary Figure S3) to allow clear visualisation of both pre-miR bands and RNA size markers.

To confirm GSK3β-enhanced pre-miR production, small RNAs (<200 nt) were isolated from HEK293T cells treated with 99021 or transfected with S^9^A-GSK3β or K^85^R-GSK3β and qPCR performed using primers targeting the miR-27a stem-loop, without amplification of pri-miRs due to size selection. It was demonstrated that 99021 treatment significantly reduced levels of pre-miR-27a (Figure 2Fi), whilst constitutively-active S^9^A-GSK3β increased pre-miR-27a levels. Dominant-negative K^85^R-GSK3β decreased pre-miR levels (Figure 2Fi), in agreement with Figure 2E and effects observed upon 99021 treatment. As expected, GSK3β-WT and -S^9^A decreased levels of pri-miR-23a27a24-2, whilst 99021 and K^85^R-GSK3β increased pri-miR-23a27a24-2 levels under the same experimental conditions (Figure 2Fii).

### GSK3β modulation alters Drosha association with pri-miRs

**Figure 3.**
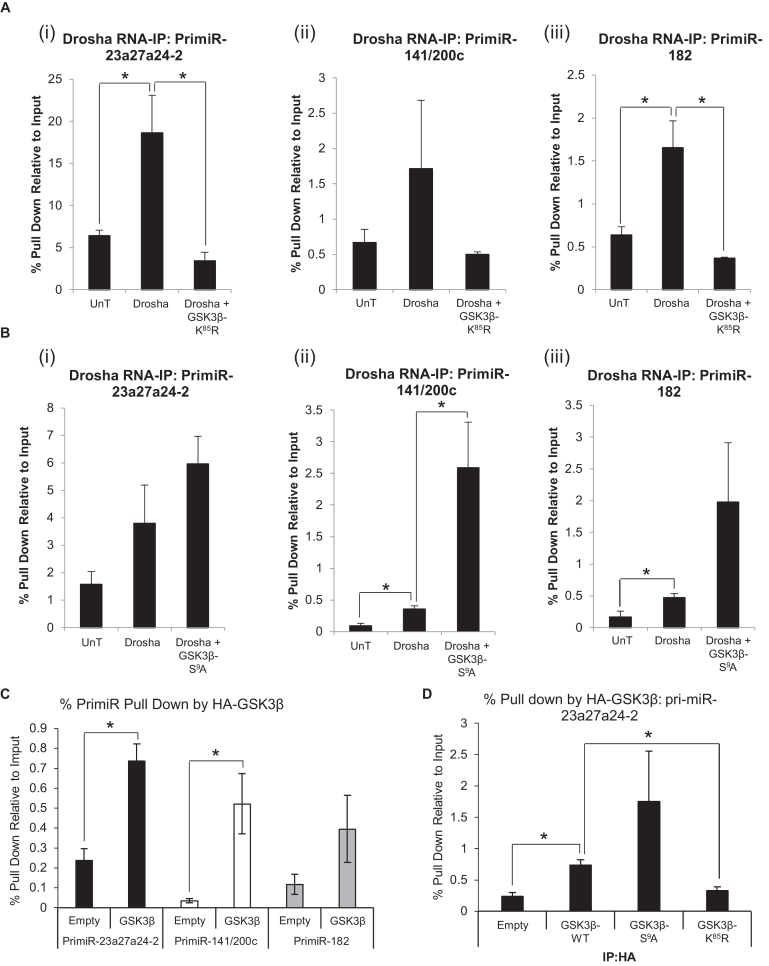
GSK3β regulates pri-MiR association with Drosha. (**A** and **B**) RNA-immunoprecipitation analysis of association of pri-miRs with Drosha in HEK293T cells ± (**A**) dominant-negative GSK3β-K^85^R or (**B**) constitutively-active GSK3β-S^9^A. Cells were transfected with Flag-Drosha ± (**A**) GSK3β-K^85^R or (**B**) GSK3β-S^9^A, then immunoprecipitated with anti-Flag antibody-bound beads and subjected to qRT-PCR analysis using (i) pri-miR-23a27a24-2, (ii) pri-miR-141/200c and (iii) pri-miR-182 primers. *Columns:* mean ± SEM for three independent experiments performed in duplicate, *P ≤ 0.05 compared to mock-transfected cells. (**C**) RNA-immunoprecipitation analysis of association of pri-miRs with wild-type GSK3β in HEK293T cells. Cells were transfected with HA-GSK3β, immunoprecipitated with anti-HA antibody-bound beads and subjected to qRT-PCR analysis using pri-miR-23a27a24-2, pri-miR-141/200c and pri-miR-182 primers. *Columns:* mean ± SEM for three independent experiments performed in duplicate, *P ≤ 0.05 compared to empty vector-transfected cells. (**D**) RNA-immunoprecipitation analysis of association of pri-miR-23a27a24-2 with constitutively-active and dominant-negative GSK3β mutants in HEK293T cells. Cells were transfected with HA-GSK3β-WT, HA-GSK3β-S^9^A or HA-GSK3β-K^85^R, immunoprecipitated with anti-HA antibody-bound beads and subjected to qRT-PCR analysis using pri-miR-23a27a24-2 primers. *Columns:* mean ± SEM for three independent experiments performed in duplicate, *P ≤ 0.05. See also Supplementary Figure S5.

To further elucidate the mechanism(s) by which GSK3β modulates Drosha activity, RNA immunoprecipitation assays were performed to evaluate the effects of GSK3β on association of Drosha with pri-miR species. HEK293T cells were transfected with a Flag-tagged Drosha expression vector and either dominant-negative or constitutively-active GSK3β mutant expression vector, followed by immunoprecipitation of the MP complex using anti-Flag antibody, RNA extraction and qRT-PCR for pri-miRs. It was demonstrated that transfection of Flag-Drosha significantly increased pri-miR-23a27a24-2 and pri-miR-182 pull-down by up to 4-fold over background binding (Figure 3Ai and iii), with a similar trend observed for pri-miR-141/200c (Figure 3Aii). Interestingly, addition of GSK3β-K^85^R to this system significantly reduced Drosha:pri-miR association to background levels (Figure 3A). These data indicate that dominant-negative GSK3β reduces global association of pri-miRs with Drosha, thereby inhibiting miR maturation. Conversely, addition of constitutively active GSK3β-S^9^A significantly enhanced association of pri-miR-141/200c with Drosha by 5-fold (Figure 3Bii) and the same trend was observed for both pri-miR-23a27a24-2 and pri-miR-182 (Figure 3Bi and iii).

To investigate whether GSK3β itself can directly bind to pri-miRs, and its involvement in MP-mediated pri-miR cleavage, HEK293T cells were transfected with HA-tagged wild-type, dominant-negative or constitutively-active GSK3β mutant expression vectors, followed by RNA immunoprecipitation using anti-HA antibody. It was found that pull-down of pri-miR-23a27a24-2 and pri-miR-141/200c was significantly increased in the presence of HA-GSKβ compared to empty vector control (Figure 3C), suggesting either that GSK3β is able to directly interact with pri-miRs, or that the presence of GSK3β in the MP (or MP-like complex) increases association of pri-miRs with this complex. Given the small fraction of pri-miR recovered in this assay, the latter may be the likelier explanation. It was also observed that constitutive activation of GSK3β increased its association with pri-miR-23a27a24-2, whilst dominant-negative GSK3β showed significantly reduced binding to pri-miR-23a27a24-4 compared to wild-type (Figure 3D). Similar effects were observed for pull down of pri-miR-141/200c and pri-miR-182 (Supplementary Figure S5). These data support our hypothesis that GSK3β enhances miR biogenesis by increasing association of Drosha with pri-miR species in a global manner, and that the presence of GSK3β within the MP complex increases pri-miR binding to the complex, possibly through direct association of GSK3β with pri-miRs.

### GSK3β alters Drosha association with MP components and interacts with DGCR8 and p72 in a RNA-dependent manner to modulate Pri-MiR to Pre-MiR processing

**Figure 4.**
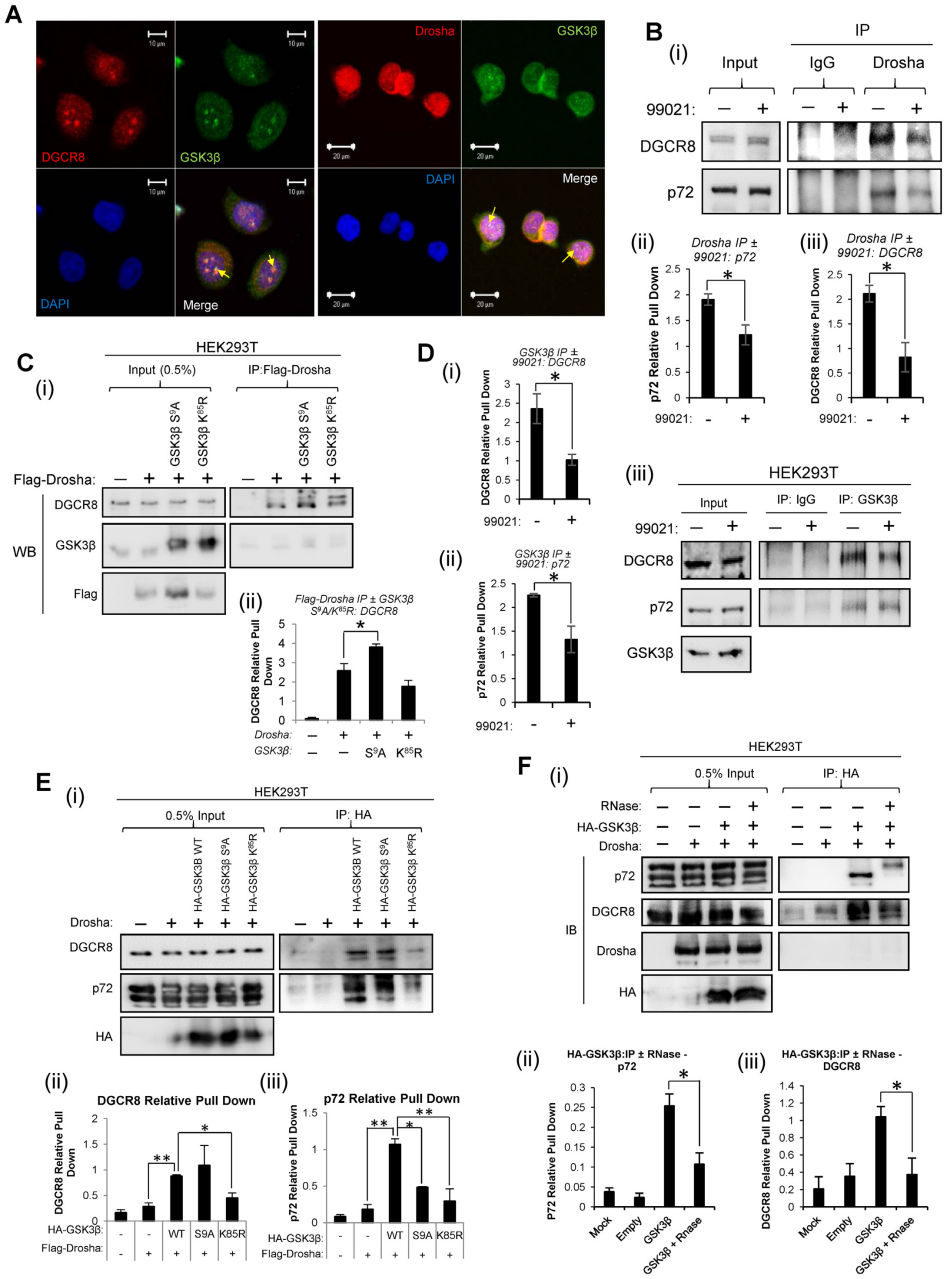
GSK3β modulates association of Drosha with DGCR8 and p72 and binds to microprocessor components to facilitate Drosha-mediated pri-miR production. (**A**) Analysis of GSK3β colocalisation with DGCR8 and Drosha in HEK293T cells by immunofluorescent antibody staining. Green, GSK3β; red, DGCR8 or Drosha; yellow, colocalisation (as indicated by arrows). Scale: as indicated by bars. Images are representative of two independent experiments, with five fields imaged per experiment. (**B**) Immunoprecipitation (IP) analysis of effects of 48 h 99021 (2 μM) treatment on interaction of endogenous Drosha with p72 and DGCR8 in HEK293T cells. 99021-treated HEK293T lysates were incubated with Drosha antibody- or rabbit IgG-bound Protein G beads and Western blotting performed for DGCR8 and p72. A representative image of three independent experiments is shown (i). Densitometry was performed using Image J software, and IP protein levels displayed relative to input (i and iii). Images of biological replicate experiments can be found in Supplementary Figure S5A. (**C**) IP analysis of interactions between exogenous Flag-Drosha and GSK3β or DGCR8. HEK293T cells were transfected with Flag-Drosha ± GSK3β-S^9^A/GSK3β-K^85^R and subject to IP using anti-Flag antibody-bound beads, followed by Western blotting for GSK3β, Flag and DGCR8. A representative image of three independent experiments is shown (i). Densitometry was performed using Image J software, and IP protein levels displayed relative to input (ii). Images of biological replicate experiments can be found in Supplementary Figure S5B. (**D**) IP analysis of effects of 48 h 99021 (2μM) treatment on interaction of endogenous GSK3β with p72 and DGCR8 in HEK293T cells. 99021-treated HEK293T lysates were incubated with GSK3β antibody- or mouse IgG-bound Protein G beads and Western blotting performed for DGCR8 and p72. A representative image of three independent experiments is shown (i). Densitometry was performed using Image J software, and IP protein levels displayed relative to input (ii and iii). (**E**) IP analysis of interactions between exogenous GSK3β and DGCR8 or p72. HEK293T cells were transfected with HA-GSK3β-WT, HA-GSK3β-S^9^A or HA-GSK3β-K^85^R and subject to IP using anti-HA antibody-bound beads, followed by Western blotting for p72, DGCR8 and HA. A representative blot of three independent experiments is shown (i). Densitometry was performed using Image J software, and IP protein levels displayed relative to input (ii and iii). (**F**) IP analysis of interactions between GSK3β and DGCR8 or p72 following RNase A treatment. HEK293T cells were transfected with HA-GSK3β-WT and subject to IP using anti-HA antibody-bound beads, followed by RNase A treatment (200μg/ml). Western blotting for p72, DGCR8, Drosha and HA was performed. A representative blot of three independent experiments is shown (i). Image J software was used for densitometry, and IP protein levels displayed relative to input (ii and iii). Images of biological replicate experiments can be found in Supplementary Figure S5C. *(A-F)* Columns represent mean ± SEM for three independent experiments. *P ≤ 0.05, **P ≤ 0.001. See also Supplementary Figure S6.

To investigate if a direct interaction between GSK3β and MP components may facilitate the GSK3β-mediated increase in Drosha activity, immunofluorescent antibody staining was performed on PC3 prostate cancer cells to assess colocalisation of endogenous proteins. GSK3β staining (green) was observed in the cytoplasm, but discrete fluorescence was also observed in the nucleus, where it showed colocalisation with both Drosha and its cofactor, DGCR8 (Figure 4A – colocalisation indicated by yellow staining). This supports the possibility of interaction between GSK3β and MP proteins. GSK3β was also found to colocalise with Drosha in the nuclei of Cos-1 and LNCaP cells following transient transfection of pMT23-HA-GSK3β and pCK-Flag-Drosha (Supplementary Figure S4). GSK3β-S^9^A and GSK3β-K^85^R demonstrated identical subcellular localisation to GSK3β-WT in both Cos-1 and LNCaP cells following transient transfection (Supplementary Figure S4).

In order to further address the possibility of interaction between Drosha and GSK3β, and to evaluate the effects of GSK3β activity on association of Drosha with other components of the MP complex, HEK293T cells were treated with 99021 for 48h and immunoprecipitation performed using Drosha antibody-bound beads. An interaction between Drosha and GSK3β was not evident (data not shown), although it is possible that such interactions are so transient as to be undetectable using this approach. However, a significant 50% reduction of Drosha association with MP components, DGCR8 and p72, was observed following 99021 treatment (Figure 4B, Supplementary Figure S6A)), indicating that GSK3β may facilitate or enhance interactions between Drosha and its MP cofactors. To corroborate these findings, HEK293T cells were co-transfected with Flag-Drosha and constitutively-active/dominant-negative GSK3β mutants and immunoprecipitation experiments performed using anti-Flag antibody. Again, no evidence was found for a physical interaction between Drosha and either GSK3β mutant under these conditions (Figure 4C, Supplementary Figure S6B middle panel - bands in IP lanes are non-specific and are observed following addition of secondary antibody only). However, it was demonstrated that constitutively active GSK3β-S^9^A significantly increased (by 50%) the association of Drosha with DGCR8, a MP cofactor that is responsible for correct orientation of Drosha on the pri-miR hairpin and is required for efficient Drosha RNase activity (Figure 4C i,ii and Supplementary Figure S6B). Addition of GSK3β-K^85^R reduced Drosha:DGCR8 binding compared to non-GSK3β mutant-transfected cells by 30%. Together these data suggest that GSK3β enhances interactions between Drosha and its vital MP cofactors DGCR8 and p72 to accelerate pri-miR processing and promote miR biogenesis. Since no evidence of direct GSK3β:Drosha interaction was seen, it was hypothesised that GSK3β may directly interact with DGCR8 and/or p72. To test this, HEK293T cells were treated with 99021 for 48 h and immunoprecipitation performed using GSK3β antibody-bound beads. Interaction of endogenous GSK3β with both DGCR8 and p72 was confirmed, and reduced by ∼40% upon treatment with GSK3β inhibitor (Figure 4D). In confirmation of this finding, DGCR8 and p72 were also demonstrated to interact with HA-tagged exogenous GSK3β (Figure 4E). Addition of dominant-negative GSK3β-K^85^R reduced these associations by 60% compared to GSK3β-WT (Figure 4E). However, whilst GSK3β-S^9^A non-significantly increased Drosha association with DGCR8 (Figure 4Eii), the presence of GSK3β-S^9^A significantly reduced interaction of Drosha with p72 (Figure 4Eiii). These data confirm binding of GSK3β to MP components, DGCR8 and p72, modulating Drosha activity and miR accumulation.

Immunoprecipitation cannot demonstrate whether interaction between GSK3β and DGCR8 and p72 is direct or occurs via another factor, such as a pri-miR. To investigate the ability of WT GSK3β to interact with the above proteins in the absence of pri-miRs, GSK3β IP experiments were performed with or without RNase A treatment. Association of GSK3β with both p72 and DGCR8 was significantly reduced by over 50% following RNase A treatment (Figure 4F, Supplementary Figure S6C). This suggests that an RNA species (mostly likely pri-miRs, given the known localisation of p72, DGCR8 and GSK3β in the MP) is required to achieve the highest extent of association of GSK3β with MP components. Minimal binding is retained in the absence of RNA, although not significantly above background levels (Figure 4F, Supplementary Figure S6C).

Taken together, these data demonstrate that GSK3β interacts with the MP cofactors DGCR8 and p72 in a pri-miR-enhanced manner to increase Drosha activity and promote miR biogenesis.

### GSK3β nuclear localisation is required for its miR biogenesis-enhancing effects

**Figure 5.**
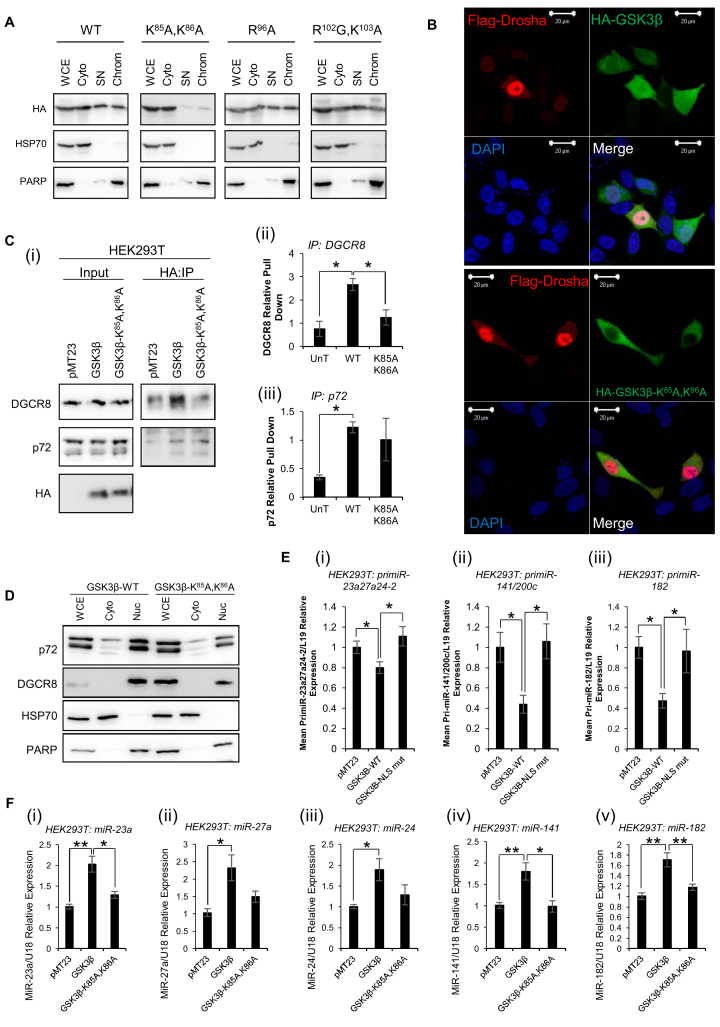
GSK3β nuclear localisation is required for its MiR biogenesis enhancing effects. (**A**) Western blot analysis of HA-GSK3β protein levels in cytoplasmic, soluble nuclear and chromatin fractions of HEK293T cells transfected with pMT23-HA-GSK3β-WT, pMT23-HA-GSK3β-K^85^A,K^86^A, pMT23-HA-GSK3β-R^96^A or pMT23-HA-GSK3β-R^102^G,K^103^A for 48 h. HSP70 and PARP were used as a cytoplasmic and nuclear controls, respectively. A representative blot of two independent experiments is shown. (**B**) Analysis of GSK3β-WT and GSK3β-K^85^A,K^86^A colocalisation with Drosha in LNCaP cells by immunofluorescent antibody staining. LNCaP cells were transfected with pCK-Flag-Drosha ± pMT23-HA-GSK3β-WT or pMT23-HA-GSK3β-K^85^A,K^86^A for 48 h, fixed and stained using antibodies against HA and Drosha. Green, HA-GSK3β; red, Drosha; yellow, colocalisation. Scale: as indicated by bars. Images are representative of two independent experiments, with four fields imaged per experiment. (**C**) IP analysis of interactions between exogenous GSK3β-WT/NLS mutant and DGCR8 or p72. HEK293T cells were transfected with pMT23, pMT23-HA-GSK3β-WT or pMT23-HA-GSK3β-K^85^A,K^86^A and subject to IP using anti-HA antibody-bound beads. Western blotting for p72, DGCR8 and HA was performed. A representative blot of three independent experiments is shown (i). Image J software was used for densitometry, and IP protein levels displayed relative to input (ii,iii). Images of biological replicate experiments can be found in Supplementary Figure S7B. Columns represent mean ± SEM for three independent experiments. *P ≤ 0.05. (**D**) Western blot analysis of p72 and DGCR8 protein levels in cytoplasmic and nuclear fractions of HEK293T cells transfected with pMT23-HA-GSK3β-WT or pMT23-HA-GSK3β-K^85^A,K^86^A for 48 h. HSP70 and PARP were used as a cytoplasmic and nuclear controls, respectively. A representative blot of two independent experiments is shown. (**E**) qRT-PCR analysis of (i) pri-miR-23a27a24-2 (ii) pri-miR-141/200c or (iii) pri-miR-182 levels in HEK293T cells transfected with pMT23-HA-GSK3β-WT or pMT23-HA-GSK3β-K^85^A,K^86^A for 48 h. L19 was used as a normalisation gene. (**F**) qRT-PCR analysis of (i) miR-23a, (ii) miR-27a, (iii) miR-24, (iv) miR-141 and (v) miR-182 levels in HEK293T cells transfected with pMT23, pMT23-HA-GSK3β-WT or pMT23-HA-GSK3β-K^85^A,K^86^A for 48 h. U18 was used as a normalisation gene. (**E** and **F**) *Columns:* mean ± SEM for three independent experiments performed in duplicate. *P ≤ 0.05, **P ≤ 0.005, See also Supplementary Figure S7.

In order to demonstrate the requirement of nuclear localisation of GSK3β for its biogenesis-promoting effects, nuclear localisation signal (NLS)-mutant HA-GSK3β constructs were generated. A putative NLS has been described between amino acids 85 and 123 of GSK3β (45). HEK293T cells were transfected with HA-GSK3β-K^85^A,K^86^A, HA-GSK3β-R^96^A and HA-GSK3β-R^102^G,K^103^A (as these have been previously shown nuclear exclusion (45)) and subcellular fractionation performed. WT GSK3β was found in both cytoplasmic and nuclear compartments (Figure 5A). HA-GSK3β-R^96^A and HA-GSK3β-R^102^G,K^103^A did not demonstrate altered localisation compared to WT GSK3β (Figure 5A). HA-GSK3β-S^9^A and HA-GSK3β-K^85^R also demonstrated similar localisation profiles to WT (Supplementary Figure S7A). In contrast, HA-GSK3β-K^85^A,K^86^A was largely excluded from soluble nuclear and chromatin-bound fractions of HEK293T cells. Nuclear exclusion of HA-GSK3β-K^85^A,K^86^A was confirmed upon immunofluorescent antibody staining of HA and Flag in LNCaP cells transfected with Flag-Drosha and HA-GSK3β-WT/ K^85^A,K^86^A (Figure 5B). To investigate the impact of GSK3β nuclear exclusion on its interactions with MP components, HEK293T cells were transfected with HA-GSK3β-WT or HA-GSK3β-K^85^A,K^86^A and IP performed using HA antibody-conjugated beads. It was demonstrated that interaction of NLS-mutant GSK3β with DGCR8 is significantly reduced to background levels compared to HA-GSK3β WT interaction (Figure 5Ci,ii and Supplementary Figure S7B). However, interaction of GSK3β with p72 is only minimally reduced by mutation of the NLS (Figure 5Ci,iii and Supplementary Figure S7B). This may be because, unlike DGCR8, p72 localises to the cytoplasm of HEK293T cells in addition to the nucleus (Figure 5D). To investigate the functional consequences of GSK3β nuclear exclusion on miR biogenesis, qPCR was performed for pri-miRs and mature miRs following transfection of HEK293T cells with HA-GSK3β WT or HA-GSK3β-K^85^A,K^86^A. As anticipated, HA-GSK3β WT significantly reduced levels of pri-miR-23a27a24-2, −141/200c and −182 (Figure 5E). This effect was abolished by addition of HA-GSK3β-K^85^A,K^86^A (Figure 5E). In corroboration of this, GSK3β-mediated increase in mature miR levels was reduced to baseline levels in the presence of GSK3β NLS mutant (Figure 5F). Taken together, these data confirm that nuclear localisation of GSK3β is required for its miR biogenesis-enhancing effects.

### GSK3β phosphorylates Drosha at S^300^ and S^302^ to modulate Drosha association with DGCR8 and p72, alter Drosha cleavage activity and regulate mature MiR levels without altering Drosha localisation

**Figure 6.**
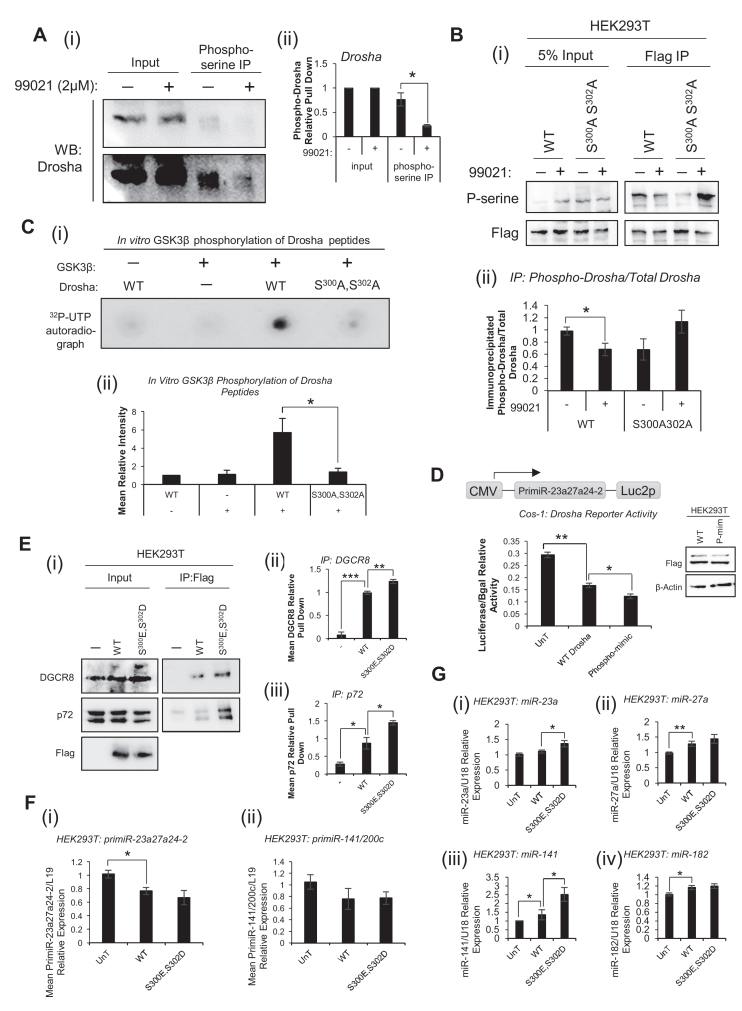
GSK3β phosphorylates Drosha at S^300^ and S^302^ to modulate Drosha association with DGCR8, alter Drosha cleavage activity and regulate mature MiR levels without altering Drosha localisation. (**A**) IP analysis of effects of 48 h 99021 (2 μM) treatment on endogenous Drosha phosphorylation. 99021-treated HEK293T lysates were incubated with anti-phosphoserine–agarose and Western blotting performed for Drosha. A representative image of three independent experiments is shown (i). Densitometry was performed using Image J software (ii,iii). (**B**) IP analysis of effects of 48 h 99021 (2 μM) treatment on phosphorylation of WT- or S^300^A,S^302^A phospho-mutant Flag-Drosha. HEK293T cells were transfected with WT- or S^300^A,S^302^A phospho-mutant Flag-Drosha, treated with 2 μM 99021 for 48 h, and subject to IP using anti-Flag antibody-bound beads, followed by Western blotting for phospho-serine and Flag. A representative image of three independent experiments is shown (i). Densitometry was performed using (ii) Image J software. (**A** and **B**) *Colums:* mean ± SEM for three independent experiments. (**C**) *In vitro* kinase assay analysis of GSK3β phosphorylation of WT or S^300^A,S^302^A-mutant Drosha. Recombinant GSK3β was incubated with 25aa peptides corresponding to WT or S^300^A,S^302^A-mutant Drosha for 60 min in the presence of (γ-32P)-ATP. Reaction products were spotted onto nitrocellulose membrane, washed and exposed to film. A representative image of three independent experiments is shown (i). Images of biological replicate experiments can be found in Supplementary Figure S8B. Densitometry was performed using Image J software (ii). *Columns:* mean ± SEM for three independent experiments. (**D**) Luciferase activity in extracts of COS-1 cells transfected with 100 ng CMV-PrimiR-23a27a24-2-GL4 vector and 0, 100 or 250 ng pCK-Flag-Drosha(WT) or pCK-Flag-Drosha(S^300^E,S^302^D) for 48 h. Luciferase was normalised for transfection efficiency (β-galactosidase activity) and mean ± SEM of three independent experiments performed in duplicate is shown. Western blotting analysis of Flag-Drosha protein levels was performed in HEK293T cells transfected with pCK-Flag-Drosha(WT) or pCK-Flag-Drosha(S^300^E,S^302^D) for 48 h to demonstrate equal transfection efficiency and expression of the two plasmids in luciferase reporter assays. (**E**) IP analysis of interactions between exogenous pCK-Flag-Drosha-WT/-S^300^E,S^302^D and DGCR8 or p72. HEK293T cells were transfected with pCK-Flag-Drosha(WT) or pCK-Flag-Drosha(S^300^E,S^302^D) for 48 h and subject to IP using anti-Flag antibody-bound beads. Western blotting for p72, DGCR8 and Flag was performed. A representative blot of three independent experiments is shown (i). Images of biological replicate experiments can be found in Supplementary Figure S8C. Image J software was used for densitometry, and IP protein levels displayed relative to input (ii,iii). (**F**) qRT-PCR analysis of (i) pri-miR-23a27a24-2 or (ii) pri-miR-141/200c levels in HEK293T cells transfected with pCK-Flag-Drosha(WT) or pCK-Flag-Drosha(S^300^E,S^302^D) for 48 h. L19 was used as a normalisation gene. (**G**) qRT-PCR analysis of (i) miR-23a, (ii) miR-27a, (iii) miR-24, (iv) miR-141 and (v) miR-182 levels in HEK293T cells transfected with pCK-Flag-Drosha(WT) or pCK-Flag-Drosha(S^300^E,S^302^D) for 48 h. U18 was used as a normalisation gene. (**F** and **G**) *Columns:* mean ± SEM for three independent experiments performed in duplicate. *P ≤ 0.05, see also Supplementary Figure S8.

Having established that GSK3β activity promotes miR biogenesis as a MP component through increasing association of Drosha with pri-miRs and cofactors, and in light of reports that Drosha is a target for GSK3β-mediated phosphorylation (34), we sought to investigate the impact of such putative post-translational modifications on Drosha's pri-miR processing activity. This will allow us to discover whether or not miR-modulatory effects of GSK3β are attributable, at least in part, to its phosphorylation of Drosha. We sought first to confirm phosphorylation of Drosha by GSK3β at S^300^ and S^302^. To this end, HEK293T cells were treated with 99021 and lysates immunoprecipitated using anti-phosphoserine antibody and subjected to immunoblotting for Drosha. Phosphorylation of Drosha was confirmed, and found to be decreased following GSK3β inhibition by 99021 (Figure 6A), indicating that Drosha is a substrate for GSK3β kinase activity. However, some phospho-Drosha was still detectable following GSK3β inhibition (Figure 6A) implying that Drosha is also a substrate for other kinases. To provide evidence for GSK3β phosphorylation of Drosha specifically at S^300^ and S^302^, HEK293T cells were transfected with WT Flag-Drosha or S^300^A^302^A phospho-mutant Flag-Drosha and treated with 99021, followed by anti-Flag immunoprecipitation and immunoblotting for phospho-serine. It was demonstrated that phosphorylation of WT Flag-Drosha is reduced following GSK3β inhibition (Figure 6Bi,ii), corroborating Drosha as a GSK3β substrate. In addition, phosphorylation of Flag-Drosha containing mutated S^300^ and S^302^ residues is reduced compared to Flag-Drosha-WT and is not lost following GSK3β inhibition (Figure 6Bii), supporting the hypothesis that GSK3β can phosphorylate Drosha at S^300^ and S^302^. Indeed, levels of phospho-Drosha are increased after 99021 treatment following ablation of S^300^ and S^302^ phosphorylation sites (Figure 6B). The differential effects of WT Flag-Drosha or S^300^A^302^A phospho-mutant Flag-Drosha are not attributable to altered Drosha localisation, since these constructs show comparable localisation profiles upon subcellular fractionation of transfected HEK293T cells (Supplementary Figure S8A).

To directly demonstrate phosphorylation of Drosha at S^300^ and/or S^302^ by GSK3β, *in vitro* kinase assays were performed, whereby recombinant GSK3β was incubated with 25aa peptides corresponding to WT or S^300^A,S^302^A-mutant Drosha in the presence of (γ-32P)-ATP. Observed phosphorylation of WT Drosha peptide in the presence of GSK3β was lost upon mutation of S^300^ and S^302^ residues (Figure 6C, Supplementary Figure S8B), confirming that GSK3β phosphorylates Drosha at either one or both of these amino acids. We next sought to demonstrate the functional consequences of GSK3β phosphorylation on Drosha function. Cos-1 cells were transfected with the previously described Drosha reporter plasmid and either WT, or phospho-mimic S^300^E,S^302^D Drosha expression vector, which has been previously used to mimic phosphorylation of Drosha at S^300^ and S^302^ (41). It was demonstrated that Drosha S^300^E,S^302^D significantly reduced luciferase activity compared to WT Drosha, indicating increased pri-miR cleavage by phospho-mimic Drosha (Figure 6D), and suggesting that GSK3β phosphorylation of Drosha increases its RNase activity. Equal expression of both Drosha constructs was confirmed by Western blotting (Figure 6D, inset). To investigate the influence of Drosha phosphorylation at S^300^ and/or S^302^ on Drosha association with its MP cofactors, HEK293T cells were transfected with Flag-Drosha WT or S^300^E,S^302^D phospho-mimic and IP performed with Flag antibody-bound beads. Phospho-mimic Drosha demonstrated significantly increased association with both DGCR8 and p72 compared to WT Drosha (Figure 6E, Supplementary Figure S8C). To assess the functional effects of phospho-Drosha on mature and pri-miRs, HEK293T cells were transfected with Flag-Drosha WT or S^300^E,S^302^D phospho-mimic and qPCR performed for pri-miR-23a27a24-2 and −141/200c, and miR-23a, −27a, −141 and −182. No significant difference in pri-miRs levels was observed between Flag-Drosha WT and S^300^E,S^302^D-transfected cells (Figure 6F), and whilst levels of miR-27a and −182 were not significantly altered in the presence of phospho-mimic Drosha (Figure 6Gii,iv), miR-23a and miR-141 levels were significantly increased in the presence of the S^300^E,S^302^D construct (Figure 6Gi,iii).

Together, these data suggest that GSK3β phosphorylates Drosha at S^300^ and/or S^302^, leading to enhanced association with p72 and DGCR8, increased Drosha RNase activity towards pri-miRs and increased levels of mature miRs.

### GSK3β-regulated MiR target proteins are increased following GSK3β inhibition

**Figure 7.**
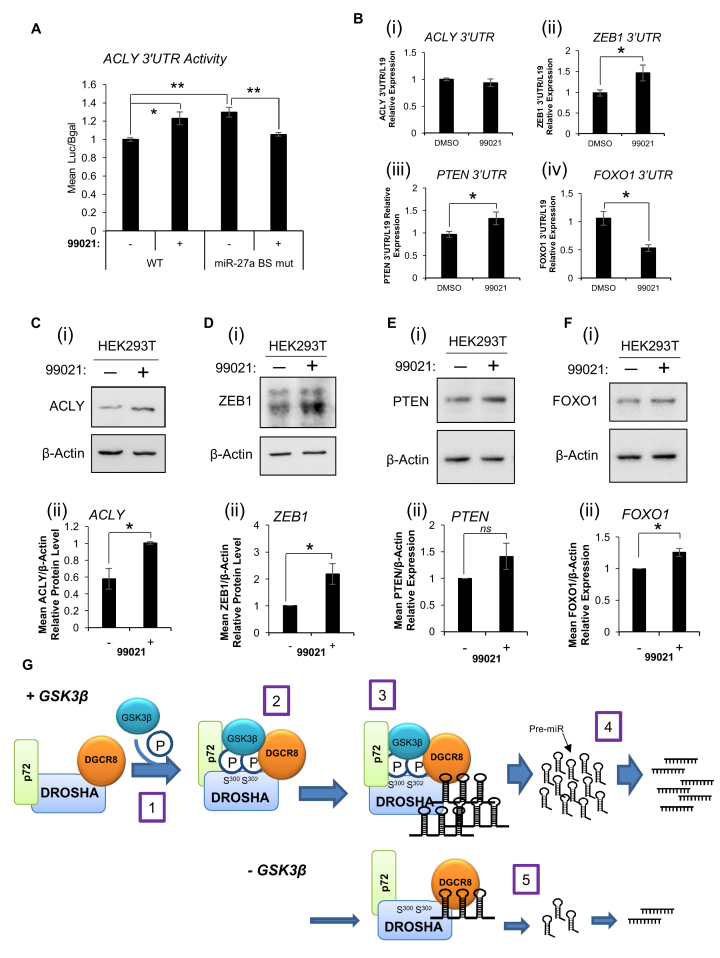
GSK3β-regulated MiR target proteins are increased following GSK3β inhibition – mechanisms for enhanced MiR biogenesis by GSK3β. (**A**) Luciferase activity in lysates of HEK293T cells transfected with pMiRTarget-ACLY-WT or pMiRTarget-ACLY-miR27a binding site (BS) mutant (for details see Supplementary Figure S9) and treated with 99021 (2 μM, 24 h). Luciferase was normalised for transfection efficiency (β-galactosidase activity) and mean ± SEM of three independent experiments performed in duplicate is shown. (**B**) qRT-PCR analysis of (i) ACLY, (ii) ZEB1, (iii) PTEN and (iv) FOXO1 3΄UTR levels in HEK293T cells treated ± 99021 (2 μM) for 72 h. L19 was used as a normalisation gene. *Columns:* mean ± SEM for three independent experiments performed in triplicate. (**C, D, E** and **F**) Western blot analysis of (**C**) ACLY, (**D**) ZEB1, (**E**) PTEN (**F**) and FOXO1 protein levels in lysates of HEK293T cells treated ± 99021 (2 μM) for 72 h. β-Actin was used as a loading control and for normalisation. Representative blots of three independent experiments are shown (i). Densitometry was performed using Image J software (ii). *Columns:* mean ± SEM for three independent experiments. *P ≤ 0.05. (**G**) A mechanism for enhanced miR biogenesis by GSK3β. GSK3β phosphorylates Drosha at S^300^ and S^302^. This does not alter levels of miR biogenesis proteins or modulate Drosha localisation but increases Drosha association with DGCR8, p72 and pri-miRs and enhances Drosha pri-miR cleavage activity, reducing pri-miR and increasing mature miR levels. GSK3β achieves these effects as a component of the Microprocessor, binding to p72 and DGCR8 in an RNA-dependent manner.

Having established that GSK3ß regulates miR biogenesis, we wished to determine whether this is likely to have downstream functional consequences. To this end, we studied the effects of GSK3β inhibition on expression of the miR targets, at the levels of target 3΄UTR activity, mRNA levels and protein levels. Using a 3΄UTR reporter construct for the miR-27a target *ACLY* (in which the *ACLY* 3΄UTR was sub-cloned 3΄ of the luciferase gene in the pMiRTarget vector), it was found that luciferase activity was significantly increased following addition of 99021 (Figure 7A). This suggests that GSK3β inhibition relieves targeting of ACLY 3΄UTR by miR-27a, presumably by preventing GSK3β-mediated upregulation of miR-27a. However, when the miR-27a binding site was mutated in this reporter construct, which abrogated the miR-27a-mediated loss of ACLY 3΄UTR activity observed for the WT construct (Supplementary Figure S9), we observed significantly higher 3΄UTR activity compared to WT 3΄UTR reporter (Figure 7A) – indicative of reduced miR-27a binding to ACLY 3΄UTR, which was additionally not increased upon 99021 treatment. This supports the hypothesis that 99021 treatment reduces miR-27a levels, relieving repression of ACLY 3΄UTR, and confirms that effects are specifically attributable to alterations in miR-27a, since no increase in 3΄UTR activity upon GSK3β inhibition was observed when the miR-27a binding site was mutated. We next examined whether 99021 treatment affected levels of the 3΄UTR of *ACLY, ZEB1, PTEN* and *FOXO1* (miR-27a, miR-141, miR-141 and miR-182/27a targets, respectively). It was demonstrated that for *ZEB1* (Figure 7Bii) and *PTEN* (Figure 7Biii) 3΄UTR levels were increased as anticipated. However, *ACLY* 3΄UTR levels were unchanged (Figure 7Bi) and *FOXO1* 3΄UTR levels decreased (Figure 7Biv) following addition of 99021. This may be due to the different mechanisms of repression of these targets by miR-27a. In cases where a miR acts via promoting translational repression, reduction of miR levels (here as a consequence of GSK3ß inhibition) would result in derepression of translation leading to increased protein levels without necessarily affecting mRNA levels. However, in cases where the miR exerts its effects via promoting trancript degradation, we would expect GSK3β inhibition to result in increased mRNA expression with a corresponding increase in protein levels. In accordance with this, both ACLY and FOXO1 protein levels were increased following addition of 99021 (Figure 7C and F), as were protein levels of ZEB1 and PTEN (Figure 7D and E). These data demonstrate that GSK3ß modulation of miR biogenesis has physiological consequences through altering protein levels of GSK3ß-regulated miR targets.

## DISCUSSION

MiRs are dysregulated in many diseases, notably cancer, where they can act as tumour suppressors or oncogenes. Recent data have given tantalising clues as to the complex signalling pathways and cascades that impinge upon miR synthesis, and it is vital to fully understand regulatory processes governing miR biogenesis, and their perturbation in disease states, in order to exploit miRs as an ‘untapped’ repository of disease biomarkers and therapeutic targets.

GSK3β is a serine/threonine protein kinase that plays a key role in signal transduction during processes such as cell cycle progression, proliferation and inflammation through phosphorylation of target proteins and shows altered activity in a number of cancers. Interestingly, GSK3β has been shown to phosphorylate Drosha at residues S^300^ and S^302^, and it has been suggested that such modifications are required for Drosha nuclear localisation (34). In addition, small-molecule inhibition of GSK3β reduced mature levels of more than 90% of miRs in mESCs, purportedly due to loss of Drosha nuclear localisation (33).

We hypothesised that GSK3β could link pro-survival signalling pathways and miR biogenesis in human somatic cells, and sought to examine the effects of GSK3β on Drosha's essential ribonuclease activity and to identify the mechanism by which GSK3β modulates miR biogenesis. We first transfected HEK293T cells with dominant-negative GSK3β-K^85^R. This decreased mature miR levels, increased pri-miR levels and reduced Drosha activity without altering Drosha protein levels or cellular localisation (Figure 1). These data suggested that inhibition of GSK3β reduces Drosha-mediated pri-miR cleavage, thereby decreasing levels of a number of different miRs. To corroborate this we used a specific small-molecule inhibitor of GSK3β, CHIR-99021 (99021), which demonstrates greater than 500-fold selectivity over closely-related kinases (22), and were able to fully replicate the results obtained with GSK3β-K^85^R: decreased mature miR levels and increased pri-miR levels, as attributable to reduced Drosha activity (Figure 1 and Supplementary Figure S1). These data further support the hypothesis that GSK3β inhibition reduces miR biogenesis. Our demonstration of similar extents of regulation for diverse miRs of different genomic contexts and subject to different modes of transcriptional regulation, when taken together with the finding that 90.4% of differentially-regulated miRs were downregulated following 99021 treatment of mESCs (33), are suggestive of widespread GSK3β modulation of miR biogenesis, although how widespread remains to be verified.

To provide additional evidence for the importance of GSK3β in global miR maturation, we employed constitutively-active GSK3β-S^9^A and observed the converse effect: an increase in Drosha activity, leading to increased mature miR levels and a loss of pri-miRs (Figure 2A–D). Taken together, these data highlight an important role for GSK3β in facilitating miR biogenesis by increasing Drosha activity towards pri-miRs. To confirm our hypothesis that GSK3β regulates miR biogenesis at the level of MP activity, *in vitro* pri-miR processing assays were performed, whereby *in vitro* transcribed pri-miR-23a27a24-2 was incubated with Flag-Drosha-containing MP immunoprecipitated from cells transfected with GSK3β mutant. Should effects of GSK3β be at the level of pri-miR processing, we anticipated pre-miR levels to be increased by GSK3β-WT and –S^9^A, and reduced by GSK3β-K^85^R. Indeed, GSK3β increased Drosha-mediated pre-miR production, an effect entirely abrogated by GSK3β-K^85^R (Figure 2E). In support of this, size-selection qPCR showed increased pre-miR levels following GSK3β-S^9^A transfection, whilst 99021 treatment significantly reduced pre-miR-27a abundance (Figure 2Fi). This was corroborated by reduced pri-miR levels in the presence of GSK3β-WT and –S^9^A, and significantly increased pri-miR-27a following GSK3β-K^85^R transfection (Figure 2Fii), in addition to enhanced Drosha reporter activity in the presence of GSK3β-S^9^A (Figure 2D). Thus, we have provided substantial evidence that GSK3β acts at the level of the MP to increase Drosha RNase activity and enhance pri-miR to pre-miR processing.

To elucidate the mechanism(s) by which GSK3β influences Drosha activity, we first examined the effect of GSK3β inhibition or activation on Drosha:pri-miR binding using RNA-IP assays. It was found that dominant-negative GSK3β significantly reduced association of Drosha with several pri-miRs, whilst constitutively active GSK3β increased it (Figure 3A and B). These data indicate that GSK3β facilitates miR biogenesis by promoting interaction between Drosha and pri-miRs. We then investigated the ability of GSK3β itself to associate with pri-miRs, and found that the presence of HA:GSK3β resulted in significantly increased anti-HA immunoprecipitation of pri-miRs with respect to controls (Figure 3C). In corroboration of this, dominant-negative GSK3β was found to significantly reduce pri-miR-23a27a24-2 immunoprecipitation with reference to the wild-type protein, whilst the constitutively-active protein increased pri-miR-23a27a24-2 pull-down compared to WT-GSK3β (Figure 3D). There are two possible interpretations of these data: firstly, that GSK3β interacts directly with pri-miRs to enhance their processing, perhaps retaining pri-miRs in the correct orientation for efficient Drosha cleavage, since GSK3β lacks RNase function; secondly, that GSK3β forms an intrinsic and important stimulatory component of a pri-miR-binding complex (probably the MP), without direct pri-miR association. The latter scenario is most likely, since GSK3β has neither been demonstrated nor predicted to contain a DNA or RNA binding motif, and alters biogenesis of all miRs assayed, regardless of level of pri-miR structure branching, number of stem-loops in pri-miR or pri-miR length, factors which would be expected to determine extent of GSK3β regulation if it bound pri-miRs directly. Interestingly, GSK3β is known to phosphorylate proteins, for example, c-Jun, close to their DNA binding domain, altering affinity for DNA (46). Thus, it is a tempting hypothesis that GSK3β additionally phosphorylates DGCR8 or p72 near their RNA binding domains to increase affinity for pri-miR substrates. Further, functional assignment of GSK3β interactors in HepG2 cells identified 24 interactors as ‘nucleic acid binding proteins’ – the largest functional group described (47). This hints at previously unexplored but important functions of GSK3β in RNA processing pathways. It also appears that GSK3β may be prerequisite for Drosha:pri-miR interaction, since pri-miR pulldown by Drosha in the presence of dominant-negative GSK3β is reduced to background levels (Figure 3A).

To provide evidence for interactions between GSK3β and MP components in the nucleus, we first performed immunofluorescent staining and found evidence for nuclear colocalisation of GSK3β with both Drosha and its cofactor, DGCR8 (Figure 4A). Although GSK3β is often considered a cytoplasmic protein, our findings are supported by the observation that 48% of GSK3β interacting proteins are localised in the nucleus of cells (47), suggestive of an important nuclear role for GSK3β. In order to investigate potential interaction between GSK3β and Drosha, IP experiments were performed. No interaction was identified between GSK3β and Drosha under our experimental conditions, although it remains possible that such an association exists but is below the detection threshold for IP, particularly given the transient nature of kinase:substrate interactions and demonstration by ourselves (Figure 6A–C) and others (34) that Drosha is phosphorylated by GSK3β. Indeed, Drosha was not identified as a GSK3β-binding protein in a recent GSK3β interactome study (47). We did, however, demonstrate that GSK3β modulation alters interactions between Drosha and its MP cofactors: IP of endogenous Drosha demonstrated significant loss of association with both DGCR8 and p72 upon 99021 treatment of HEK293T cells (Figure 4B), and constitutively active GSK3β was shown to increase association of Drosha with DGCR8 (Figure 4C). Interestingly, DGCR8 has been identified as a phospho-protein, with phosphorylation at 23 sites demonstrated to increase its stability, resulting in a pro-growth miR profile (48). Although JNK and ERK proteins were identified as candidate kinases, the authors examined only a fraction of the kinome and additional potential DGCR8-phosphorylating proteins are not known. Given GSK3β's primary function as a kinase, it is an attractive hypothesis that GSK3β could phosphorylate DGCR8 to increase its stability, promoting its increased interaction with Drosha and thus enhanced pri-miR cleavage. Indeed IP assays both with endogenous and HA-tagged GSK3β confirmed a previously undescribed interaction with DGCR8, which was significantly diminished in the presence of 99021 (Figure 4D) or the dominant-negative mutant (Figure 4E). Interestingly, GSK3β also demonstrated a novel interaction with p72 (DDX17) (Figure 4D and E), a DEAD-box MP cofactor that acts as a specificity protein for processing distinct subsets of miRs, and is important for miR maturation (49). This provides additional evidence for the importance of GSK3β in miR biogenesis, and is of further interest since p72 has been demonstrated to alternatively splice GSK3β mRNA, increasing cellular levels of the shorter, more catalytically active GSK3β isoform-1 (50). This provides another level of complexity to regulation of miR maturation, whereby p72 not only regulates Drosha cleavage directly, but may also alter the equilibrium between the two GSK3β isoforms in order to regulate MP activity. Reciprocal regulation is also possible via GSK3β phosphorylation of p72, and/or the extent of the p72:GSK3β interaction itself. GSK3β has been demonstrated to interact with other miR-regulatory and RNA-binding proteins: for example, DDX1 and DDX21, which are closely-related to p72, and the heterogeneous nuclear ribonucleoproteins hnRNPA2B1 and hnRNPK, whose family member, hnRNPA1, is a key MP component (47). Additionally, FUS and p68, further components of the MP complex, were identified as participants in a cluster with GSK3β (47), adding further weight to the argument that GSK3β facilitates miR biogenesis as a component of the MP complex, although modulation of Dicer activity by GSK3β has not be ruled out. Providing further evidence of the importance of GSK3β in pri-miR processing, its association with both DGCR8 and p72 was lost upon RNase treatment of HEK293T cells (Figure 4F), suggesting requirement of an RNA species, likely a pri-miR, for association of GSK3β with MP components and enhancement of Drosha activity.

Having demonstrated interaction of GSK3β with p72 and DGCR8 that is reduced in the absence of RNA, we sought to illustrate the requirement of GSK3β nuclear localisation for its miR biogenesis promoting activity. To this end, we tested a number of previously reported NLS mutants that had demonstrated nuclear exclusion in HEK293 and HeLa cells (45). HA-GSK3β-K^85^A,K^86^A, but not –R^96^A nor R^102^G,K^103^A, was found to be excluded from the nucleus of HEK293T cells, as demonstrated both by subcellular fractionation and immunofluorescent microscopy (Figure 5A and B). Subsequent analyses showed that interaction of HA-GSK3β-K^85^A,K^86^A with DGCR8 is reduced compared to WT-GSK3β (Figure 5Ci,ii). Interestingly, however, interaction with p72 was not altered by NLS mutation (Figure 5Ci,iii). This is likely to be because p72 localises to the cytoplasm in addition to the nucleus (Figure 5D), thus its interaction with NLS mutant GSK3β may be representative of cytoplasmic binding. Functionally, pri-miR levels were increased (Figure 5E) and mature miR levels reduced (Figure 5F) in the presence of HA-GSK3β-K^85^A,K^86^A compared to the WT protein, suggesting nuclear localisation of GSK3β is required for optimal Drosha cleavage of pri-miR substrates.

Given that GSK3β has been demonstrated to phosphorylate Drosha at S^300^ and S^302^ (34), we sought to clarify the impact of such post-translational modifications on Drosha RNase activity and to establish whether this is the mechanism by which GSK3β alters Drosha:DGCR8, Drosha:p72 and Drosha:pri-miR interactions. We first used IP methods to demonstrate that levels of phospho-Drosha are reduced upon 99021 treatment (Figure 6A), suggesting that GSK3β can phosphorylate Drosha *in vitro*. These data confirm previous reports describing Drosha phosphorylation by this kinase (34,41). To provide evidence that such phosphorylation occurs at S^300^ and S^302^, exogenous WT- or S^300^A,S^302^A-Flag-Drosha were immunoprecipitated and levels of serine-phosphorylated Drosha examined. It was demonstrated that, in corroboration of the above discussed data, phosphorylation of WT Flag-Drosha is significantly decreased upon GSK3β inhibition, and that phosphorylation of S^300^A,S^302^A-Flag-Drosha is considerably reduced compared to WT Drosha (Figure 6B). Interestingly, phosphorylated WT-Drosha is still detectable upon GSK3β inhibition (Figure 6A and B), suggesting that Drosha is also serine-phosphorylated by other kinases. Further, levels of phospho-Drosha are increased following 99021 treatment of cells transfection with S^300^A,S^302^A-Flag-Drosha, raising the possibility that inhibition of GSK3β derepresses activity of other kinases that phosphorylate Drosha at other sites. Of note, our demonstration that abrogating GSK3β-mediated phosphorylation of Drosha at S^300^ and S^302^ (Supplementary Figure S8A) does not alter its cellular localisation contrasts with previous data illustrating loss of Drosha nuclear localisation upon 99021 treatment of mESCs (33), and loss of nuclear Drosha in GSK3β^−/−^ MEFs compared to WT cells (34). This may be due to differences in mechanisms of GSK3β regulation of miR biogenesis between the mouse embryonic cell lines used in prior studies (33,34), and human HEK293T cells employed for our experiments, or effects of GSK3β manipulation independent of kinase activity at S^300^/S^302^.

Definitive evidence that GSK3β phosphorylates Drosha at S^300^ and/or S^302^ has been provided by *in vitro* kinase assays: GSK3β can phosphorylate WT, but not S^300^A,S^302^A mutant Drosha peptide *in vitro* (Figure 6C). In terms of the impact of GSK3β phosphorylation of S^300^/S^302^ on Drosha activity, luciferase activity of a pri-miR-23a27a24-2-specific Drosha reporter construct was reduced following addition of S^300^E,S^302^D phospho-mimic Drosha compared to WT-Drosha (Figure 6D), indicating enhanced Drosha cleavage. Further, phospho-mimic Drosha showed significantly enhanced interaction with its cofactors, DGCR8 and p72, as compared to WT-Drosha (Figure 6E), suggesting that GSK3β-mediated phosphorylation of Drosha at S^300^ and S^302^ increases miR biogenesis by increasing Drosha:cofactor interactions to promote pri-miR cleavage. This is further supported by significantly increased levels of miR-23a and −141 in HEK293T cells transfected with phospho-mimic Drosha compared to WT (Figure 6G). Minimal effects observed on pri-miR and mature miR-27a and −182 levels may be attributable to maximal Drosha activity in HEK293T cells transfected with WT Flag-Drosha, such that Drosha activity towards pri-miRs cannot be significantly elevated upon addition of Flag-Drosha-S^300^E,S^302^D. Together, these data support the hypothesis that GSK3β is the kinase responsible for Drosha phosphorylation at S^300^ and S^302^, leading to enhanced Drosha RNase activity and miR accumulation.

To demonstrate the physiological relevance of GSK3β regulation of miR biogenesis, we examined 3΄UTR activity and protein levels of targets of miRs demonstrated above to be regulated by GSK3β. We demonstrated that 3΄UTR activity of ACLY (a target of GSK3β-upregulated miR-27a) was increased upon 99021 treatment (Figure 7A), which reduces miR-27a levels (Supplementary Figure S1D). In addition, protein levels of ACLY (miR-27a target – Figure 7C), ZEB1 (miR-141 target – Figure 7D), PTEN (miR-141 target – Figure 7E) and FOXO1 (miR-182 and −27a target, Figure 7F) were significantly increased following addition of 99021, indicating derepression by their targeting miRs due to loss of GSK3β-enhanced miR biogenesis. These data demonstrates that GSK3β modulation of miR biogenesis has physiological consequences by altering protein levels of GSK3β-regulated miR targets.

It is clear that GSK3β constitutes an important link between multiple pro-survival signalling pathways and miR biogenesis. Perturbation of GSK3β activity may disrupt the highly-regulated process of miR maturation and may play a role in disease pathology, carcinogenesis or maintenance of an oncogenic phenotype. In addition, alterations to the phosphorylation status of GSK3β in response to various stimuli may act as a rheostat that regulates miR biogenesis and the ratio of pri- to mature miRs by modulating Drosha activity. Further work is required to confirm whether GSK3β regulation of miR maturation is a global effect, and to establish whether GSK3β phosphorylates or interacts with other MP components in addition to Drosha, DGCR8 and p72. It is possible that additional auxiliary factors are required to confer specificity of GSK3β regulation of miR biogenesis, and that GSK3β may contribute to a pre-MP holoenzyme, associating with DGCR8, p72 and other factors to assemble pre-MP configuration commensurate with Drosha binding. It will be very informative to further examine the GSK3β-regulated miRnome in human cells to see if the miR subset identified is enriched for particular cellular functions or processes, as this could have important implications for GSK3β as a drug target in cancer, where it displays contrasting and ambiguous roles.

In conclusion, these data are the first to identify an unexpected and entirely novel important role for GSK3β in post-transcriptional regulation of miR biogenesis as a component of the MP and RNase cofactor: GSK3β binds to p72 and DGCR8 in the nuclear MP in an RNA-dependent manner, leading it to phosphorylate Drosha at S^300^ and S^302^ (Figure 6A–C). This does not alter levels of miR biogenesis proteins or modulate Drosha localisation, but increases Drosha association with DGCR8, p72 and pri-miRs, and enhances Drosha RNase and pri-miR cleavage activity, reducing pri-miR and increasing mature miR levels, likely due to stabilisation of MP configuration. This has profound implications for GSK3β as a drug target in cancer and other pathologies, and for understanding miR biogenesis as a highly complex and stringently-controlled process on which a multitude of vital signalling cascades converge.

## Supplementary Material

Supplementary Data
