# Supplementary material for: A novel role for GSK3β as a modulator of Drosha microprocessor activity and MicroRNA biogenesis
Source: Nucleic Acids Res. 2016 Oct 23;45(5):2809–28. doi: 10.1093/nar/gkw938 (PMC5389555; doi:10.1093/nar/gkw938)
Supplement: Supplementary Data [file gkw938_Supp.zip › nar-02779-a-2015-File009.pdf]

Fletcher et al

Supplementary Material

Supplementary Methods

RNA Immunoprecipitation

4x10cm dishes of HEK293T cells were transfected with pCK-Flag-Drosha ± pMT23-c-Myc-GSK3β-S^9A or pMT23-HA-GSK3β-K^{85}R mutant GSK3β expression vector using the calcium phosphate method [48]. 48h post-transfection, cells were washed in ice-cold PBS and pooled per condition. 3ml lysis buffer (50mM Tris-HCl pH7.5, 150mM NaCl, 1% Triton-X, 1mM EDTA, 160U/ml RNAsin, 0.5mM DTT plus protease and phosphatase inhibitors) was used to resuspend pelleted cells from 4x10cm dishes. Each lysate was passed three times through a 25G needle and once through a 27G needle and incubated on ice for 15min. Lysates were centrifuged at 16,000*g* for five minutes at 4˚C. Supernatant was removed and 10% retained for input. 80µl of anti-Flag affinity gel (Sigma) per lysate was washed three times in 2ml TBS, resuspended to original volume and added to the prepared lysate. Lysate:anti-Flag bead mixes were rotated overnight at 4˚C. Anti-Flag beads were then pelleted and washed x3 5min with wash buffer (50mM Tris-HCl pH7.5, 300mM NaCl, 5mM $MgCl_2$, 0.05% NP40 plus protease and phosphatase inhibitors) at 4˚C. Beads were then reconstituted with 100µl DNase solution (Qiagen) and incubated at room temperature for 15min. Beads were then treated with 0.2µg/ml proteinase K in proteinase K buffer (100mM Tris-HCl pH 7.5, 150mM NaCl, 12.5mM EDTA and 2% SDS) for 20min at room temperature with rotation. RNA was then extracted from beads using Trizol LS (Ambion) according to manufacturer's instructions and qRT-PCR performed for pri-miRs.

Flag-Drosha Subcellular Fractionation

HEK293T cells previously treated with 99021 (2µM), transfected with pMT23-GSK3β-WT/ S^9A/ K^{85}R/ K^{85}A,K^{86}A/ R^{96}A/ R^{102}G,K^{103}A, or transfected with pCK-Flag-WT/S^{300}A,S^{302}A or plasmids in 10cm dishes were pelleted by centrifugation for 3 minutes at 1,500x*g* and washed twice by resuspension in PBS. Cells were resuspended in buffer A (10mM HEPES, 10mM KCl, 1.5mM $MgCl_2$, 0.34M sucrose, 10% glycerol 1mM DTT, 0.1mM PMSF and 5µl/ml proteinase inhibitor cocktail, pH 7.4) such that 200µl buffer A contained $3x10^6$ cells. Twenty µl 1% Triton-X was added to cell suspensions, which were incubated on ice for 5 minutes. Cells were centrifuged at 1,300x*g* at 4°C for 4 minutes. Supernatant (cytoplasmic fraction) was removed and combined with 200µl 2x SDS buffer. Cell pellets were washed once in 200µl buffer A, pelleted as above, and resuspended in 200µl buffer B (3mM EDTA, 0.2mM EGTA, 1mM DTT and 5µl/ml proteinase inhibitor cocktail). Samples were incubated on ice

for 30 minutes, followed by centrifugation at 1,700x*g* for 4 minutes at 4°C. Supernatant (containing soluble nuclear fraction) was removed and combined with 200μl 2x SDS buffer and stored on ice until required. Insoluble chromatin pellets were washed once in 200μl buffer B and pelleted by centrifugation at 1,700x*g* at 4°C for 4 minutes. Supernatant was disgarded and the chromatin pellet resuspended in 200μl 2x SDS. For whole cell extracts (WCEs), $3x10^6$ cells were resuspended in 200µl PBS, to which 200µl 2xSDS was added. All fractions were boiled for 10-15 minutes then cooled on ice prior to Western blotting.

Immunoprecipitation

3x10cm dishes of HEK293T cells per immunoprecipitation reaction were transfected with Flag-Drosha (WT, $S^{300}QS^{302}Q$ or $S^{302}A$) ± HA-tagged GSK3β-$S^9A/K^{85}R$ as appropriate using the calcium phosphate method for 48h. Cells were washed with ice-cold PBS and lysed in 600µl ice-cold IP lysis buffer (50mM Tris-HCl pH7.4, 150mM NaCl, 1mM EDTA, 1% Triton-X, 10µM PMSF and 5µl/ml of proteinase inhibitor cocktail) per 10cm dish and incubated on ice for 20min with agitation. Lysates were pooled for each IP reaction condition and centrifuged at 12,000*g* for 10min at 4˚C. Supernatant was removed into a pre-chilled tube and 10% removed and combined with an equal volume of 2xIP sample loading buffer (125mM Tris-HCl pH6.8, 4% SDS, 20% glycerol, 0.004% bromophenol blue) for inputs. Each pooled lysate was pre-cleared with 150µl ms IgG agarose beads (Sigma A0919) for 30min at 4˚C with rotation. Beads were pelleted by centrifugation at 14,000*g* for 10min at 4˚C and lysates removed and kept on ice. 60µl of resuspended anti-Flag M2 agarose beads (Sigma) or EZView anti-HA agarose beads (Sigma) as appropriate was pelleted and washed x3 with TBS per lysate and resuspended to original volume in IP lysis buffer. 60µl of washed resuspended beads was then added to each pre-cleared lysate and incubated overnight at 4˚C with rotation. Beads were pelleted by centrifugation and washed 2x 5min and 1x 15min at 4˚C with TBS. An optional RNase A treatment was performed by addition of RNase A at a final concentration of 200µg/ml for 15min at 4°C. Upon removal of the final wash, beads were resuspended in an equal volume of 2x IP sample loading buffer, boiled for three minutes and chilled on ice. Agarose beads were then pelleted and supernatant removed for Western blotting.

Immunofluorescent Antibody Staining of Exogenous Proteins

Cos-1 cells on coverslips were transfected with Flag-Drosha and either GSK3β-S^9A or GSK3β-$K^{85}R$ for 24-48h. Cells were washed in 1xPBS and fixed with 1% formaldehyde in PBS for 10 minutes. Cells were permeabilised with 0.1% Triton X for 10 minutes at room temperature, washed in PBS and blocked with 10% goat serum in PBS for 1h. Primary antibodies (Rb anti-Drosha and Ms anti-GSK3β were diluted 1/100 in 10% goat serum and

added to cells for 1h at room temperature. Following washing with PBS, secondary antibodies (Alexa Fluor 488 Goat anti-Mouse and 594 Goat anti-Rabbit SFX kits, Invitrogen) were diluted 1/200 in 10% goat serum and added to cells for 1h at room temperature in the dark. Cells were washed briefly in PBS and coverslips were mounted onto glass slides using DAPI-containing Vectashield mounting solution. Staining was visualised using a Zeiss LSM510 confocal microscope.

Fletcher et al

Supplementary Table

Primer Name	Sequence (5' → 3')
Drosha For	CCCTCCGGGCTATTCTCAC
Drosha Rev	TGGTCATCATAGTGTTTCAGCCT
ACLY 3'UTR For	GTACAGGCACCGAAGACCAA
ACLY 3'UTR Rev	ACAGCAGGTAGCAGAGCAAA
ZEB1 3'UTR For	AGCAAGACCTGTGTGCTGTA
ZEB1 3'UTR Rev	GCCACACATTGGATGAAGGC
PTEN 3'UTR For	CTGCAGAAAGACTTGAAGGCG
PTEN 3'UTR Rev	GCTGTGGTGGGTTATGGTCT
FOXO1 3'UTR For	ATGGCTTGGTGTCTTTCTTTTCT
FOXO1 3'UTR Rev	TGTGGCTGACAAGACTTAACTCAA
Pre-miR-27a For	CTGAGGAGCAGGGCTTAGCTG
Pre-miR-27a Rev	TGGGGGGCGGAACTTAGCCAC
L19 For	GCGGAAGGGTACAGCCAA
L19 Rev	GCAGCCGGCGCAAA

Table S1. Nucleic acid sequences of Drosha and L19 primers used for SYBR Green qRT-PCR.
