# Supplementary material for: A novel role for GSK3β as a modulator of Drosha microprocessor activity and MicroRNA biogenesis
Source: Nucleic Acids Res. 2016 Oct 23;45(5):2809–28. doi: 10.1093/nar/gkw938 (PMC5389555; doi:10.1093/nar/gkw938)
Supplement: Supplementary Data [file gkw938_Supp.zip › nar-02779-a-2015-File010.pdf]

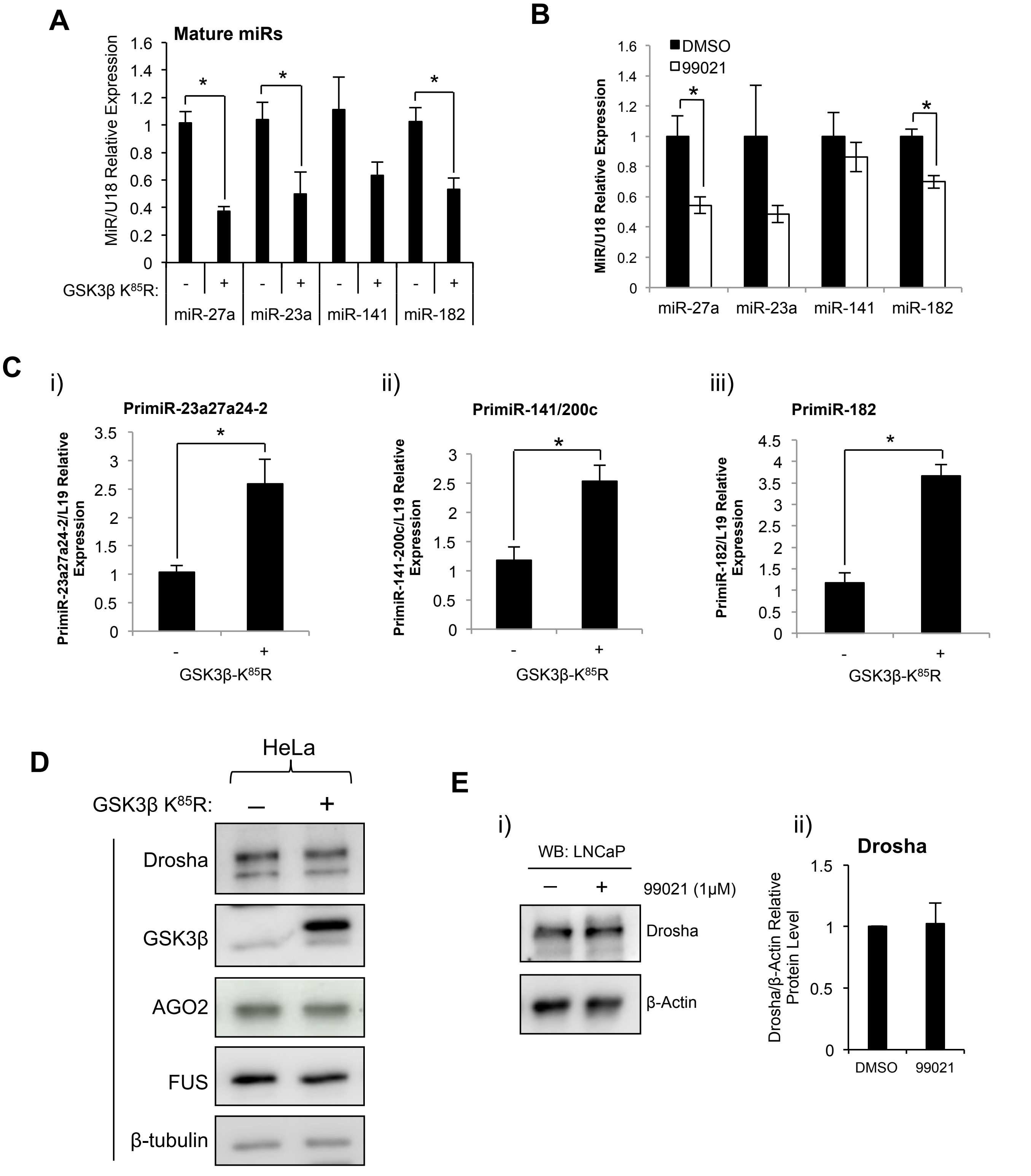

Figure S1: Effects of Dominant-Negative GSK3β-$K^{85}R$ and the GSK3β Inhibitor, 99021, on MiR Biogenesis in Cancer Cells. *(A)* qRT-PCR analysis of miR-182, miR-23a, miR-27a and miR-141 levels in LNCaP cells transfected with pMT23-HA-GSK3β($K^{85}R$) for 48h.*(B)* qRT-PCR analysis of miR-141, miR-182 and miR-23a levels in LNCaP cells treated with 1μM 99021 for 48h. *(A,B)* U18 was used as a normalisation gene. *(C)* qRT-PCR analysis of primiR-23a27a24-2 *(i)*, primiR-141/200c *(ii)* and primiR-182 *(iii)* expression from HeLa cells transfected with pMT23-HA-GSK3β($K^{85}R$) for 48h. L19 was used as normalisation gene. *(A,B,C) Columns:* mean ± SEM for three independent experiments performed in triplicate. * $P \leq 0.05$. *(D)* Western blot analysis of Drosha, AGO2 and FUS protein levels in HeLa cells transfected with pMT23-HA-GSK3β($K^{85}R$) for 48h. β-Tubulin was used as a loading control. *(E)* Western blot analysis of Drosha levels in LNCaP prostate cancer cells treated with 1μM 99021 for 48h *(i)*. β-Tubulin was used as a loading control and densitometry performed using Image J software *(ii)*. *(D,E)* Representative blots of three independent experiments are shown. See also Figure 1.

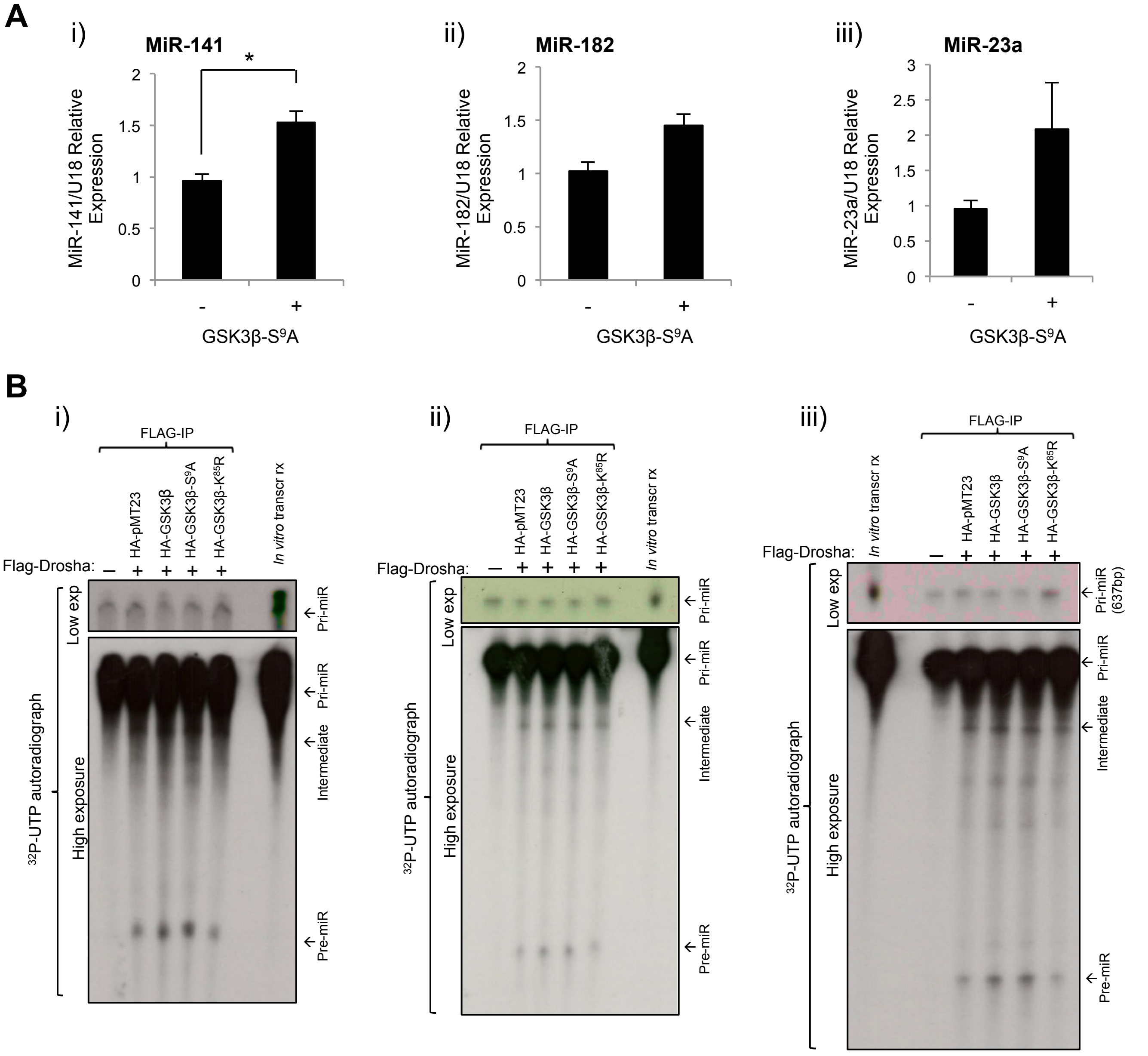

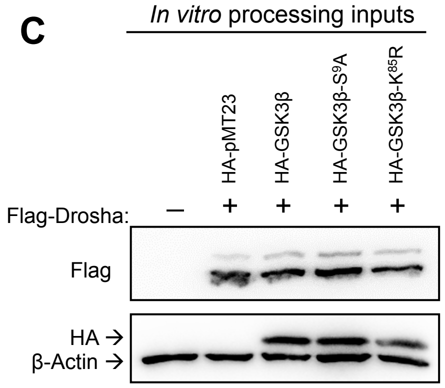

Figure S2: Effects of Constitutively-Active GSK3β-S^{9}A on Mature MiR Levels in Cancer Cells. *(A)* qRT-PCR analysis of miR-141 *(i)*, miR-182 *(ii)* and miR-23a *(iii)* levels in HeLa cells transfected with pMT23-HA-GSK3β(S^{9}A) for 48h. U18 was used as a normalisation gene. *Columns:* mean ± SEM for three independent experiments performed in triplicate. * $P \leq 0.05$. *(B) In vitro* Drosha-mediated pri-miR processing assay analysis of pre-miR levels following incubation of *in vitro* transcribed radio-labelled pri-miR-23a27a24-2 with Microprocessor complex immunoprecipitated from HEK293T cells transfected with Flag-Drosha ± GSK3β-WT, -S^{9}A or -K^{85}R for 48h. Products of *in vitro* processing reactions were resolved on a 6% acrylamide:urea gel and exposed to film. Three additional independent experiments are shown *(i,ii,iii).* For *(iii)*, the Decades Markers System (ThermoFisher) was used to produce radio-labelled RNA size markers which were resolved alongside experimental samples. RNA marker sizes are as indicated. Uncropped images of *Biii,* and *in vitro* processing products resolved on 12.5% acrylamide:urea gel are shown in Fig S3. *(C)* Western blotting analysis of Flag-Drosha and HA-GSK3β levels was performed on HEK293T lysates used for *in vitro* processing assay to confirm equal input of Microprocessor complex. β-Actin was used as a loading control. See also Figure 2 and S3.

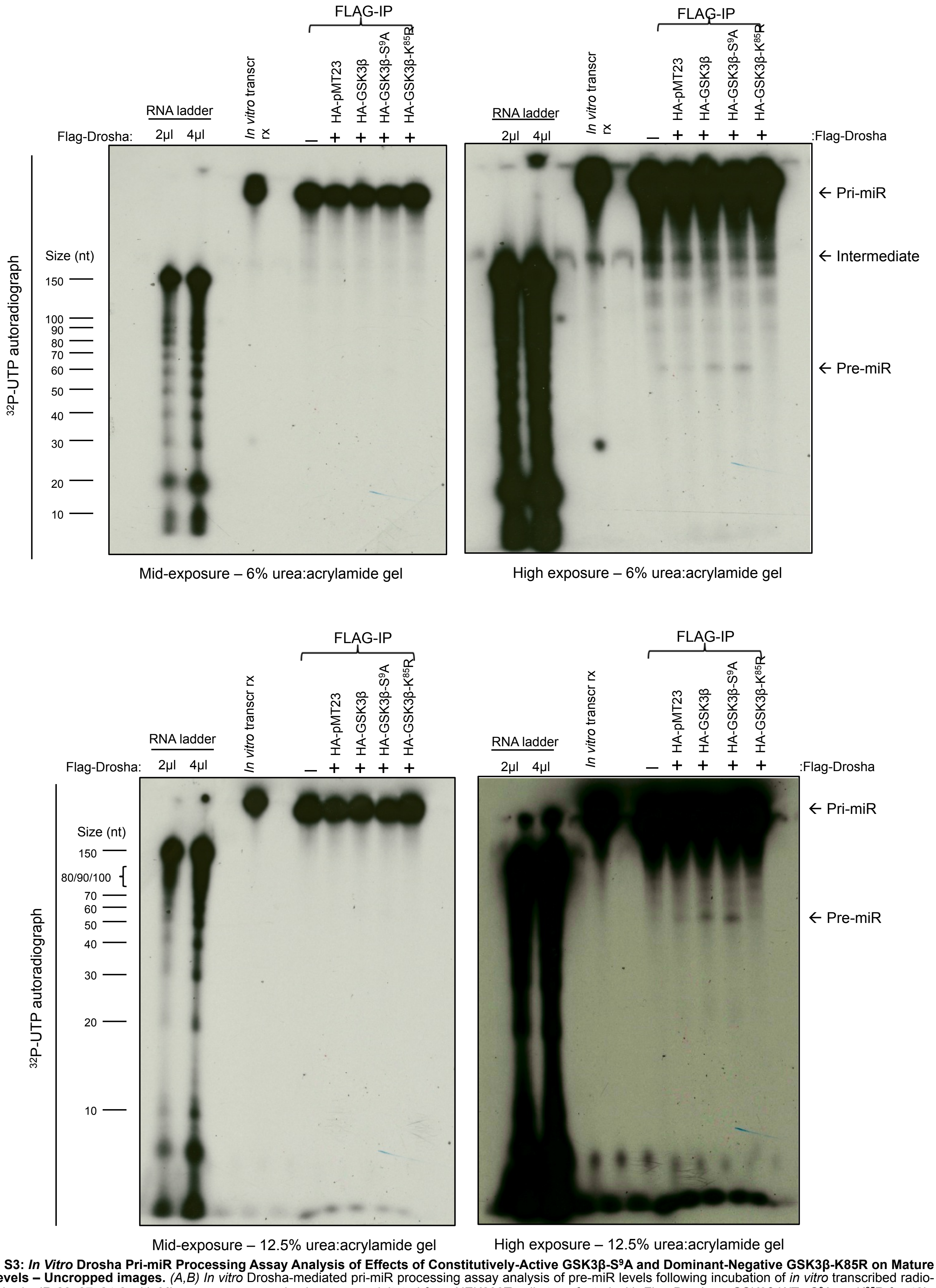

Figure S3: *In Vitro* Drosha Pri-miR Processing Assay Analysis of Effects of Constitutively-Active GSK3β-S⁹A and Dominant-Negative GSK3β-K85R on Mature MiR Levels – Uncropped images. *(A,B) In vitro* Drosha-mediated pri-miR processing assay analysis of pre-miR levels following incubation of *in vitro* transcribed radio-labelled pri-miR-23a27a24-2 with Microprocessor complex immunoprecipitated from HEK293T cells transfected with Flag-Drosha ± GSK3β-WT, -S⁹A or -K⁸⁵R for 48h. Products of *in vitro* processing reactions were resolved on a 6% *(A)* and 12.5% *(B)* acrylamide:urea gel and exposed to film. The Decades Markers System (ThermoFisher) was used to produce radio-labelled RNA size markers which were resolved alongside experimental samples. RNA marker sizes are as indicated. Uncropped images of mid *(i)* and high *(ii)* exposure are shown to provide good visualisation both of pre-miR bands and RNA markers. See also Fig S2Biii and Fig 2.

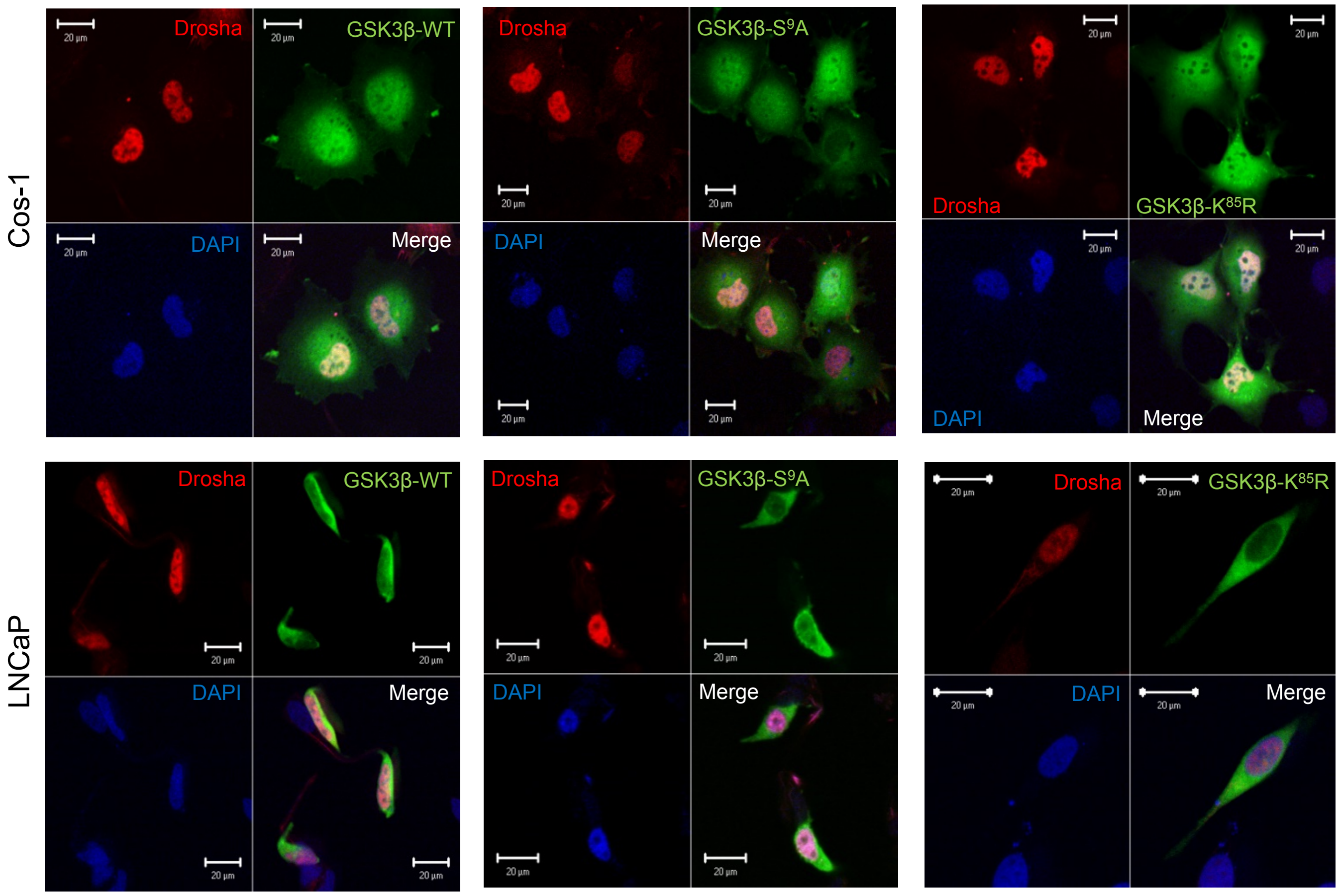

Figure S4 Subcellular Localisation of GSK3β and Drosha in Cos-1 and LNCaP Cells. Immunofluorescent antibody staining was performed on Cos-1 (top panel) or LNCaP (bottom panel) cells transfected with Flag-Drosha and WT, S^9A or $K^{85}R$ GSK3β. Slides were counterstained with DAPI and imaged by confocal microscopy. Green: GSK3β; red: Drosha. Scale: as indicated by bars. See also Figures 2 and 3.

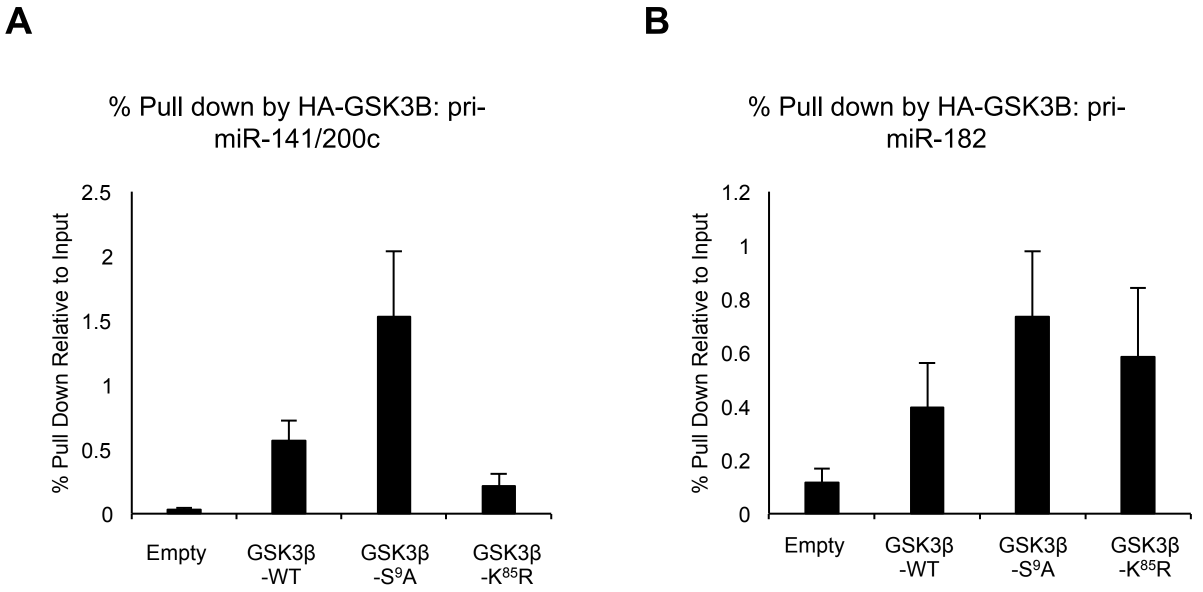

Figure S5: GSK3β Immunoprecipitates Pri-miR-141/200c and Pri-miR-182. RNA-immunoprecipitation analysis of association of *A)* pri-miR-141/200c and *B)* pri-miR-182 with constitutively-active (S^9A) and dominant-negative ($K^{85}R$) GSK3β mutants in HEK293T cells. Cells were transfected with HA-GSK3β-WT, HA-GSK3β-S^9A or HA-GSK3β-$K^{85}R$, immunoprecipitated with anti-HA antibody-bound beads and subjected to qRT-PCR analysis using pri-miR-23a27a24-2 primers. *Columns:* mean ± SEM for three independent experiments performed in duplicate, * $P \leq 0.05$.

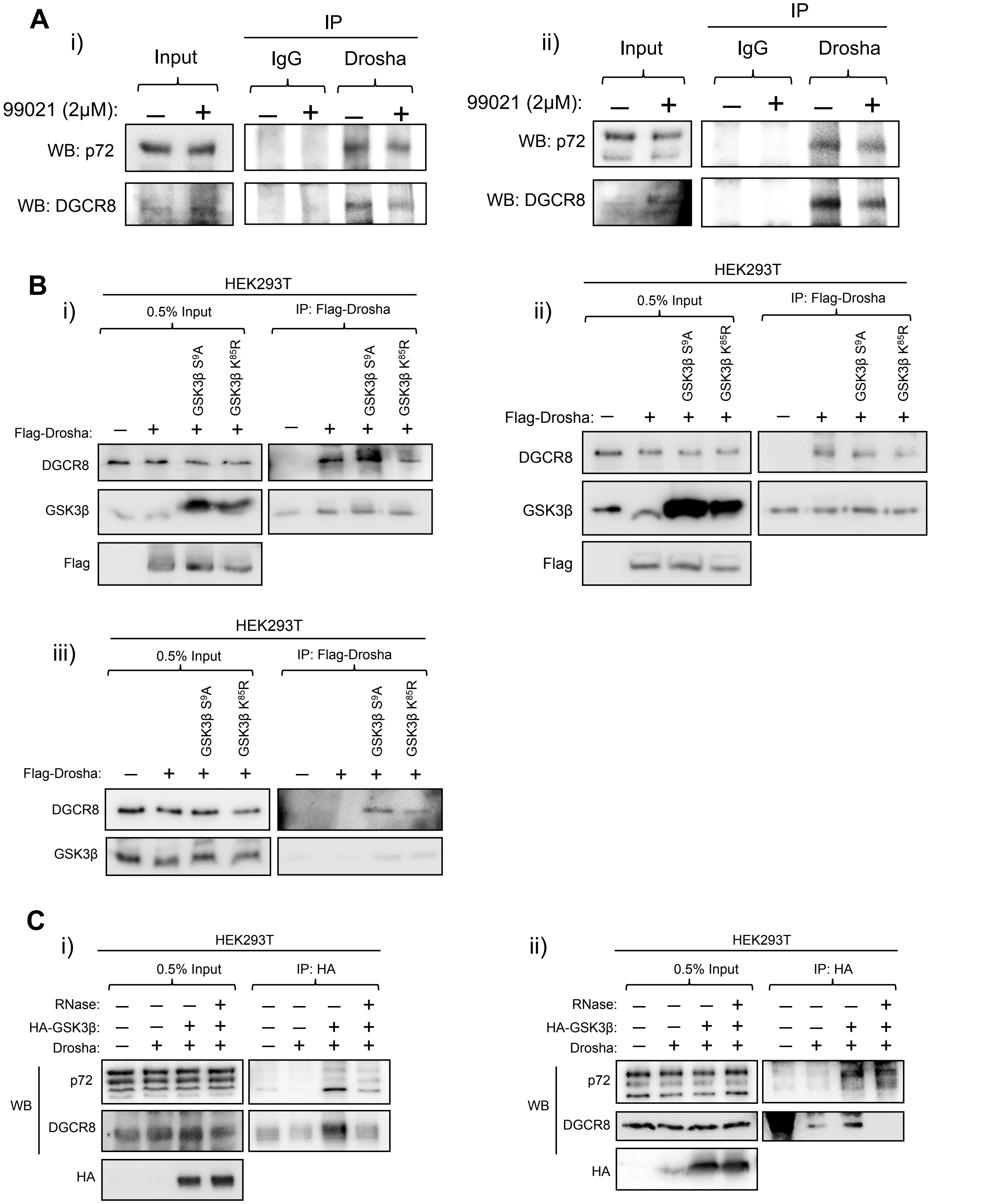

Figure S6: GSK3β Modulates Drosha Interaction with MP Components and Interacts with p72 and DGCR8 in an RNA-Dependent Manner. *(A)* Immunoprecipitation (IP) analysis of effects of 48h 99021 (2μM) treatment on interaction of endogenous Drosha with p72 and DGCR8 in HEK293T cells. 99021-treated HEK293T lysates were incubated with Drosha antibody- or rabbit IgG-bound Protein G beads and Western blotting performed for DGCR8 and p72. Two additional independent experiments are shown *(i,ii)*. *(B)* IP analysis of interactions between exogenous Flag-Drosha and GSK3β or DGCR8. HEK293T cells were transfected with Flag-Drosha ± GSK3β-S^9A/GSK3β-$K^{85}R$ and subject to IP using anti-Flag antibody-bound beads, followed by Western blotting for GSK3β, Flag and DGCR8. Three additional independent experiments are shown *(i,ii)*. *(C)* IP analysis of interactions between GSK3β and DGCR8 or p72 following RNase A treatment. HEK293T cells were transfected with HA-GSK3β-WT and subject to IP using anti-HA antibody-bound beads, followed by RNase A treatment (200μg/ml). Western blotting for p72, DGCR8, Drosha and HA was performed. Two additional independent experiments are shown *(i,ii)*. See also Fig 4.

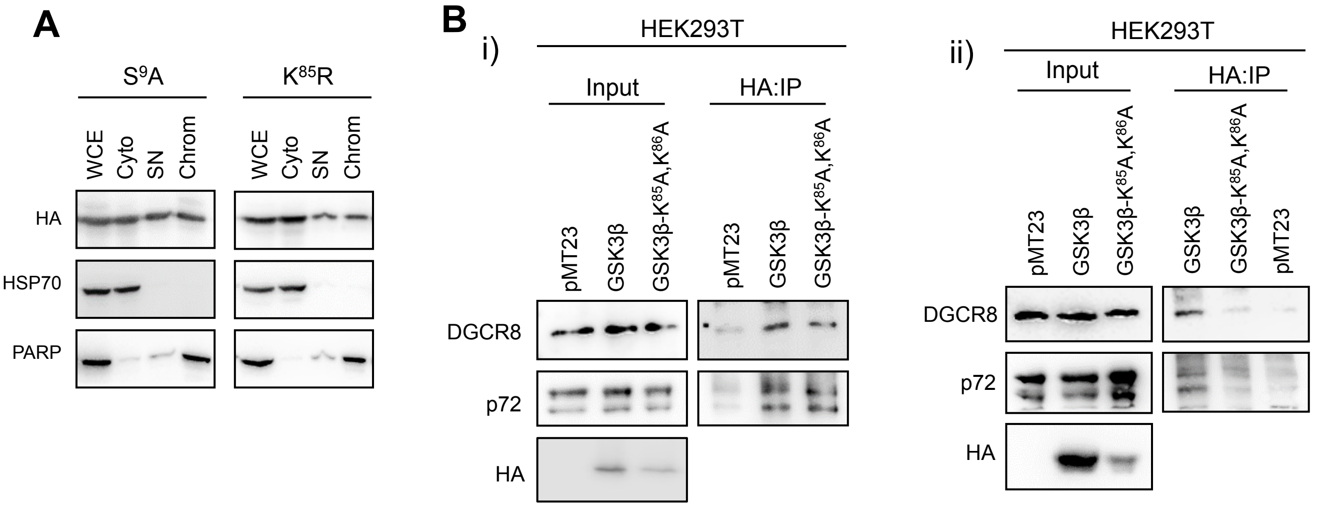

Figure S7: Subcellular Localisation of HA-GSK3β-S⁹A and –K⁸⁵R and Effects of GSK3β NLS Mutation on Interaction with DGCR8 and p72. *(A)* Western blot analysis of HA-GSK3β protein levels in cytoplasmic, soluble nuclear and chromatin fractions of HEK293T cells transfected with pMT23-HA-GSK3β-$S^{9}A$ or pMT23-HA-GSK3β-$K^{85}R$ for 48h. HSP70 and PARP were used as a cytoplasmic and nuclear controls, respectively. A representative blot of two independent experiments is shown. *(B)* IP analysis of interactions between exogenous GSK3β-WT/NLS mutant and DGCR8 or p72. HEK293T cells were transfected with pMT23, pMT23-HA-GSK3β-WT or pMT23-HA-GSK3β-$K^{85}A$,$K^{86}A$ and subject to IP using anti-HA antibody-bound beads. Western blotting for p72, DGCR8 and HA was performed. Two additional independent experiments are shown *(i,ii).* Note that in *Bii,* IP lane order is different from input lane order.

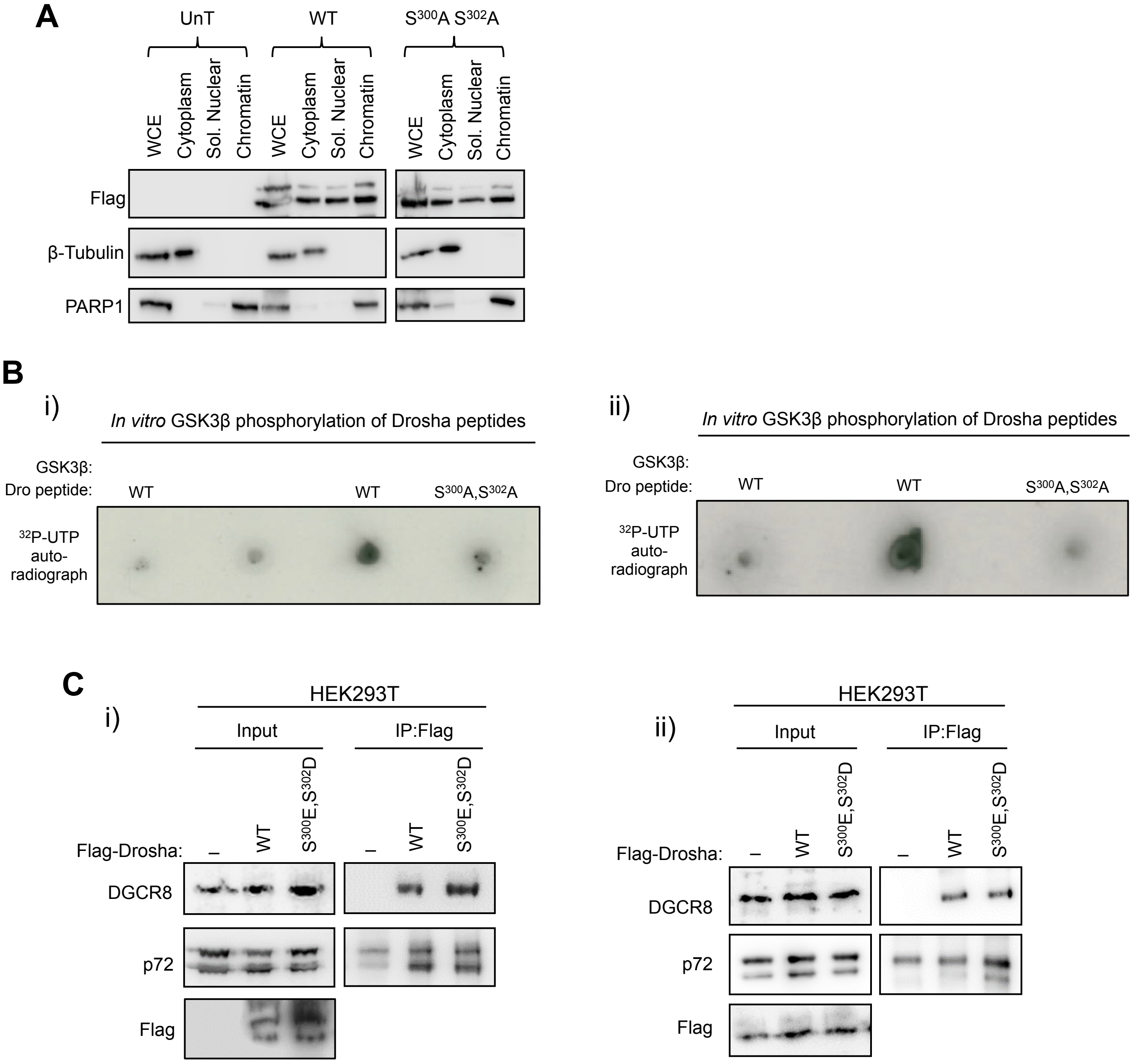

Figure S8: Mutation of Candidate Residues for GSK3β Phosphorylation does not Alter Drosha Localisation, GSK3β Phosphorylates Drosha at S^{300} and/or S^{302} and Phosphorylation of Drosha at S^{300} and/or S^{302} Enhances its Interactions with Microprocessor Components. *(A)* Western blot analysis of Flag-Drosha protein levels in cytoplasmic, soluble nuclear and chromatin fractions of HEK293T cells transfected with pCK-Flag-Drosha WT or pCK-Flag-Drosha $S^{300}A,S^{302}A$ for 48h. β-tubulin and PARP1 were used as a cytoplasmic and nuclear controls, respectively. A representative blot of two independent experiments is shown. *(B) In vitro* kinase assay analysis of GSK3β phosphorylation of WT or $S^{300}A,S^{302}A$-mutant Drosha. Recombinant GSK3β was incubated with 25aa peptides corresponding to WT or $S^{300}A,S^{302}A$-mutant Drosha for 60min in the presence of [γ-32P]-ATP. Reaction products were spotted onto nitrocellulose membrane, washed and exposed to film. Two additional independent experiments are shown *(i,ii)*. *(C)* IP analysis of interactions between exogenous pCK-Flag-Drosha-WT/-$S^{300}E,S^{302}D$ and DGCR8 or p72. HEK293T cells were transfected with pCK-Flag-Drosha(WT) or pCK-Flag-Drosha($S^{300}E,S^{302}D$) for 48h and subject to IP using anti-Flag antibody-bound beads. Western blotting for p72, DGCR8 and Flag was performed. Two additional independent experiments are shown *(i,ii).*

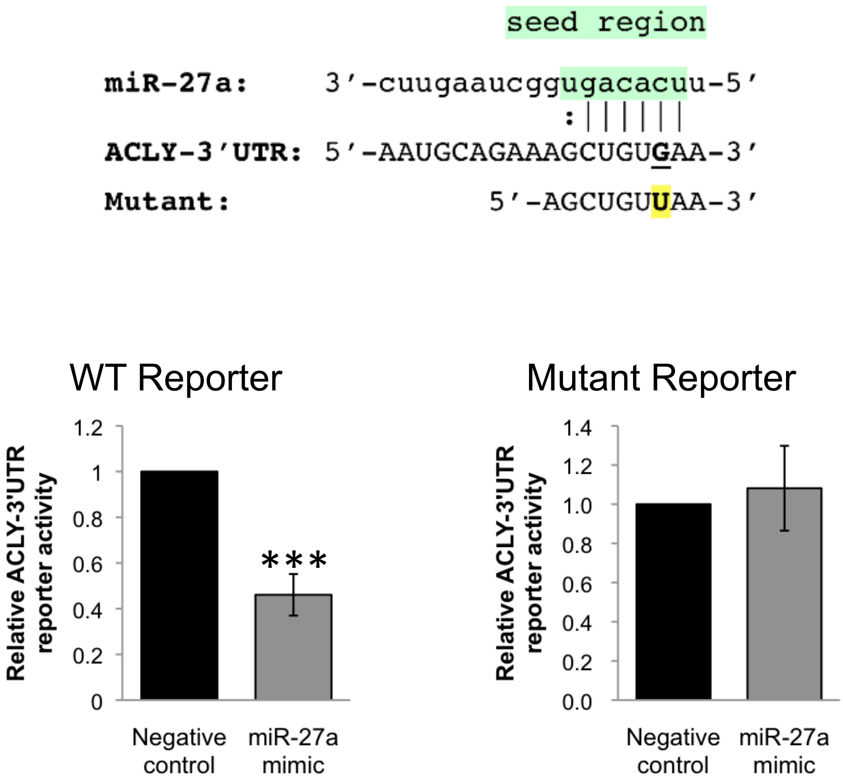

Figure S9: Mutation of ACLY 3'UTR MiR-27a Seed Region Binding Site Abrogates MiR-27a-Mediated Loss of ACLY 3'UTR Activity. *(A)* Diagram illustrating binding of miR-27a to ACLY 3'UTR and nucleotides mutated in ACLY-3'UTR miR-27a BS (binding site) mutant. *(B)* Luciferase activity in lysates of PC3 prostate cancer cells transfected with pMiRTarget-ACLY-WT or pMiRTarget-ACLY-miR27a binding site (BS) mutant alongside 10nM negative control or miR-27a mimic for 24h. Luciferase activity was normalised for transfection efficiency (β-galactosidase activity) and mean ± SEM of three independent experiments performed in triplicate is shown. ***P < 0.001.
